# Supplementary material for: Knowledge and attitudes of Implementation Support Practitioners—Findings from a systematic integrative review
Source: PLoS One. 2022 May 11;17(5):e0267533. doi: 10.1371/journal.pone.0267533 (PMC9094539; doi:10.1371/journal.pone.0267533)
Supplement: S1 Appendix — Implementation Support Skills–Findings from a Systematic Integrative Review. (PDF) [file pone.0267533.s001.pdf]

Electronic Results Addendum (ERA): Implementation Support Skills – Findings from a Systematic Integrative Review

Detailed Search Strategy

The following search strategies were used to identify publications for this review:

1. Systematic searches of nine electronic databases (ASSIA, CINAHL, Criminal Justice Abstracts, ERIC, Family and Society Studies Worldwide, Medline, PsycInfo, Scopus, and SocIndex) using a search string that combined the term "implementation" with terminology describing different support behaviors and specific implementation support role labels (S1 in Table ERA1). A key aim of this approach was to filter out literature that was not focused on active implementation support activities and instead centered on e.g. knowledge translation in general. Additionally, research-related search terms (S2 in Table ERA1) were used to limit searches to literature focused on the uptake of evidence, rather than the uptake of e.g. non-evidenced innovations. This search string was tailored to the requirements of the different databases. Table ERA1 displays the search string used with PsycInfo (OVID); all other search strings can be made available upon request to the author. Electronic databases were searched on February 26, 2019. No limitations were defined for publication dates.

Table ERA1: Search String Used with the PsycInfo/OVID Database

#	Searches
**S1**	(implement* adj3 support) OR (implement* adj3 coach*) OR (implement* adj3 speciali*) OR (implement* adj3 expert*) OR (implement* adj3 team) OR (implement* adj3 facilitat*) OR (implement* adj3 consult*) OR (implement* adj1 train) OR (implement* adj1 educat*) OR (implement* adj1 course) OR (knowledge adj3 broker*) OR (intermediar* adj3 organi$ation) OR purveyor* OR purveyour* OR (technical adj3 assist*).mp
**S2**	('research finding*' or 'research result*' or 'scientific knowledge' or 'scientific finding*' or 'evidence-informed practice' or 'evidence informed practice' or 'evidence-based practice' or 'evidence based practice' or 'research to practice' or 'research-to-practice' or 'knowledge to action' or 'knowledge-to-action' or 'science-based' or 'science based' OR 'research-based' OR 'research based' or 'science-informed' OR 'science informed or 'research-informed' or 'research informed' OR 'knowing-doing' OR 'knowing doing' OR 'science practice' OR 'science-practice').mp
**S3**	**S1 AND S2**

2. To ensure that potentially relevant publications commissioned by organizations active in the field of implementation support could be included in the review, searches of 42 different organizational websites were conducted. These websites were selected based on the research teams' knowledge about intermediary and other organizations with an interest in implementation. In searching their websites, the regular website search function and/or subsites clearly labeled as listing e.g. "resources," "publications," "research" or comparable materials were used. Searches of websites (listed in Table ERA2) were conducted between March 18 and April 30, 2019.
3. A targeted call for publications from experts known to the research team as working within the field of implementation support, e.g. as researchers, consultants or in similar roles, was conducted to identify publications under review or in press – and any other relevant article that might not be covered through other searches. Of the 22 experts contacted by email, 17 responded. E-mail exchanges took place in March, April and May 2019.
4. An open call for publications disseminated through a newsletter, a website and social media, all of which were administered by the European Implementation Collaborative (EIC). The newsletter was sent out on March 29, 2019 to 700+ recipients, who were invited to submit resources within four weeks. It was then followed up by multiple reminders posted through the EIC's twitter account.
5. Reference checks of all included studies identified through the above strategies.

Table ERA2: Websites Searched for Grey Literature

#	Organization (hyperlinked)	Date searched
**1**	Agency for Healthcare Research and Quality (U.S.)	***18.3.2019***
**2**	Alliance for Useful Evidence (U.K.)	***18.3.2019***
**3**	The Annapolis Coalition (U.S.)	***30.4.2019***
**4**	The Annie E. Casey Foundation (U.S.)	***19.3.2019***
**5**	The Bridgespan Group (U.S.)	***19.3.2019***
**6**	The Center for Implementation- Dissemination of Evidence-Based Practices Among States (IDEAS – U.S.)	***10.4.2019***
**7**	Centre for Effective Services (IRL)	***19.3.2019***
**8**	Centre for Evidence & Implementation (AUS)	***19.3.2019***
**9**	Child & Family Evidence Based Practice Consortium (U.S.)	***19.3.2019***
**10**	The Colebrooke Centre for Evidence and Implementation (U.K.)	***19.3.2019***
**11**	Consortium for Implementation Science (U.S.)	***25.3.2019***
**12**	Early Childhood TA Center (U.S.)	***25.3.2019***
**13**	Education Endowment Foundation (U.K.)	***25.3.2019***
**14**	EPIS Center (U.S.)	***25.3.2019***
**15**	European Implementation Collaborative (EU)	***25.3.2019***
**16**	The Health Foundation (U.K.)	***25.3.2019***
**17**	Institute of Education Sciences – What Works Clearinghouse (U.S.)	***3.4.2019***
**18**	iS2 (AUS)	***3.4.2019***
**19**	The Joanna Briggs Institute (AUS)	***3.4.2019***
**20**	Judge Baker Children's Centre (U.S.)	***3.4.2019***
**21**	Mathematica Policy Research (U.S.)	***3.4.2019***
**22**	mdrc (U.S.)	***10.4.2019***
**23**	National TA Center for Children's Mental Health (U.S.)	***10.4.2019***
**24**	The National Implementation Research Network (U.S.)	***10.4.2019***
**25**	The National Institute for Health and Care Excellence (U.K.)	***10.4.2019***
**26**	National Institutes of Health (U.S.)	***10.4.2019***
**27**	National TA Center on Transition (U.S.)	***10.4.2019***
**28**	Nuffield Foundation (U.K.)	***15.4.2019***
**29**	Office of the Assistant Secretary for Planning and Evaluation (U.S.)	***15.4.2019***
**30**	Ontario Centre of Excellence for Child and Youth Mental Health (CAN)	***15.4.2019***
**31**	The PEW Charitable Trusts (U.S.)	***15.4.2019***
**32**	Research in Practice (U.K.)	***15.4.2019***
**33**	The SISEP Center (U.S.)	***15.4.2019***
**34**	The Social Care Institute for Excellence (SCIE) (U.K.)	***15.4.2019***
**35**	TAC - Technical Assistance Collaborative (U.S.)	***30.4.2019***
**36**	TA Center on PBIS (U.S.)	***23.4.2019***
**37**	The Institute for Innovation and Implementation (U.S.)	***23.4.2019***
**38**	UK Implementation Society (U.K.)	***23.4.2019***
**39**	Washington State Institute for Public Policy (U.S.)	***23.4.2019***
**40**	William T. Grant Foundation (U.S.)	***23.4.2019***
**41**	The World Bank (INT)	***23.4.2019***
**42**	The World Health Organization (INT)	***23.4.2019***

Table ERA3: Inclusion and Exclusion Criteria

	INCLUDED	EXCLUDED
**Study designs**	Any primary study design, including, e.g.:<br>• Randomised, controlled studies<br>• Quasi-experimental studies<br>• Pre-post evaluations<br>• Case studies	• Systematic reviews of any study designs<br>• Theoretical / conceptual studies<br>• Expert commentary<br>• Opinion pieces<br>• Conference proceedings<br>• Books / book chapters<br>• Editorials<br>• Other publications that are / report 'non-studies'<br>• Dissertations / theses
**Population**	• The target population(s) of the study are implementation specialists.	• The target population(s) of the study are current or future *implementation researchers* or other non-ISPs.
**Sectors**	• Health care (including mental health)<br>• Social care / social welfare (including child welfare, aged care, labour market services, crime and justice, family services etc.)<br>• Education (including ECE, primary, secondary and high school services)	• IT<br>• Environmental services<br>• Any other service sector not listed in the left column.
**Content**	• The study focuses on supporting the implementation of a **research-supported program, practice or policy**<br>**AND**<br>• the study involves a person or a team or an intermediary organization providing implementation support to others<br>**AND**<br>• the study provides information about / conceptualises / assesses implementation know how / skills / capacity<br><br>Examples of interventions that could be of relevance:<br>• Implementation training<br>• Implementation coaching<br>• Technical assistance<br>• Implementation coaching<br>• Implementation facilitation<br>• Implementation policies<br>• Implementation mentorship	• Studies focused on the specific knowledge, skills, capacities required to implement e.g. specific *clinical interventions*<br>○ → e.g. a study examining skills required to implement trauma-informed practice would be excluded<br>○ → a study of a community of practice aimed at building practitioners' capacity to develop domestic violence prevention capacity would be excluded<br>○ → a study of a 'community of practice' aimed at building practitioners' implementation knowledge / skills would be included<br><br>• Studies focused on factors that support successful practice-science / community-university partnerships<br><br>• Studies focused on online solutions only (repositories providing electronic implementation resources and nothing else)
**Geography**	• High income countries<br>• Low / middle income countries	No exclusion based on geography
**Languages**	• English<br>• German<br>• Danish<br>• Swedish<br>• Norwegian	Any other language not listed

Figure ERA1: Flow of Studies Through the Review Process

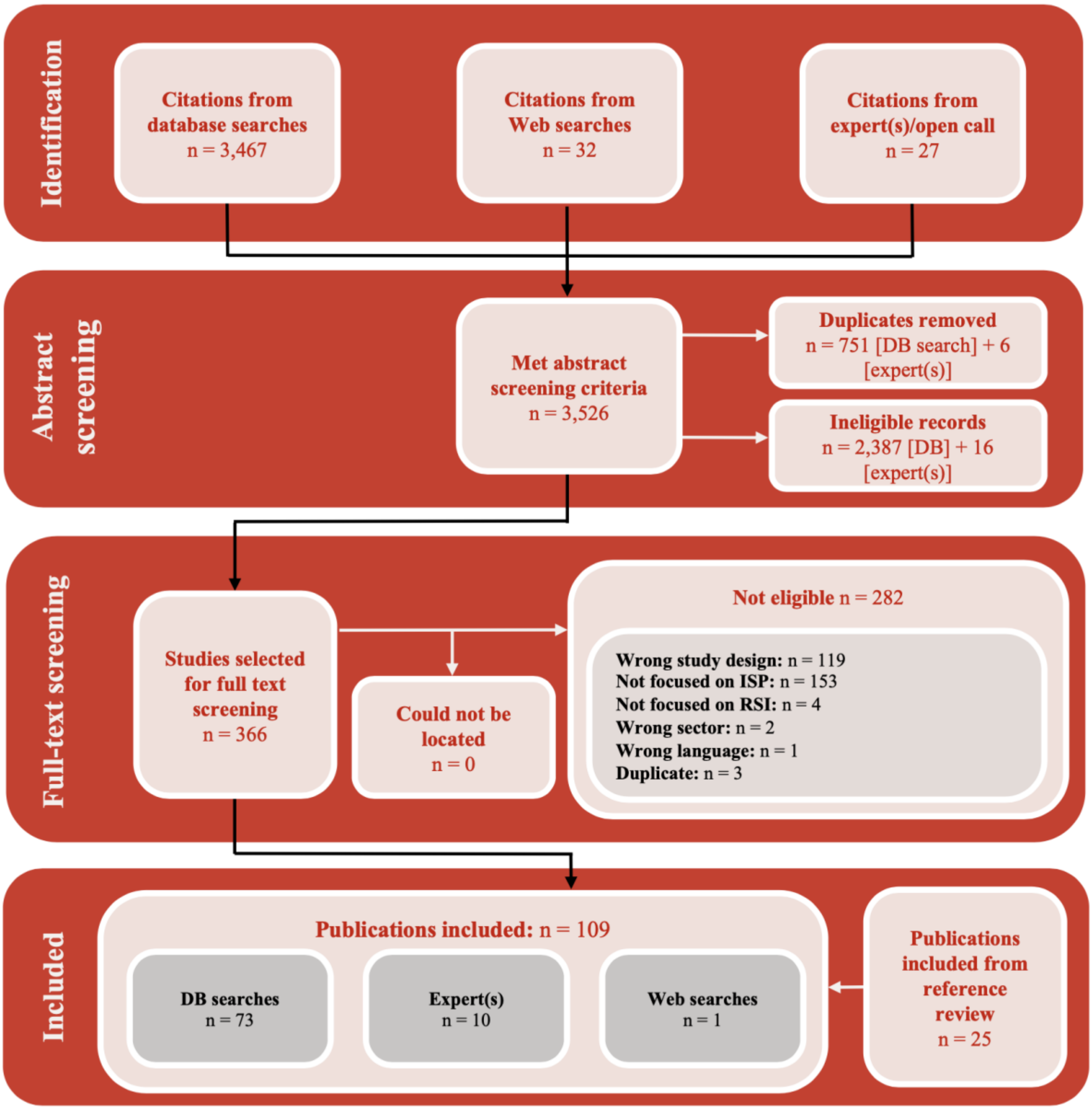

Table ERA4: Coding Examples

**Publication & text excerpt**	**Code(s) assigned**	**Code definition** (Powell et al., 2015)
Rosella (2018), p. 30: "The KT approach began with the identification of relevant needs and the cultivation of partnerships with decision-makers who agreed to joint engagement in the use of DPoRT and its outputs."	Conduct local needs assessment	Collect and analyze data related to the need for the innovation
Ritchie (2017), p. 5: "In preparation for helping sites implement PC-MHI, the facilitators began engaging clinic leaders and provided information about PC-MHI programs as well as what to expect during the facilitation process."	Identify and prepare champions	Identify and prepare individuals who dedicate themselves to supporting, marketing, and driving through an implementation, overcoming indifference or resistance that the intervention may provoke in an organization
Hurlburt (2014), p.: "Members of the seed team maintain a central liaison role between the EBP developer and other actors at multiple levels within the service system so that issues, decisions, and adaptations can be negotiated during initial experimentation with implementation."	• Promote network weaving<br>• Provide ongoing consultation	• Identify and build on existing high-quality working relationships and networks within and outside the organization, organizational units, teams, etc. to promote information sharing, collaborative problem-solving, and a shared vision/goal related to implementing the innovation<br>• Provide ongoing consultation with one or more experts in the strategies used to support implementing the innovation

Table ERA5: Distribution of Implementation Strategies by ISP Role

Implementation Strategy	Coach (N=8)	Consultant (N=21)	Facilitator (N=22)	Imp. Team (N=4)	Intermediary (N=3)	Knowledge Broker (N=10)	Mix (N=5)	Non-specified (N=5)	Other (N=9)	TA Provider (N=12)	TOTAL (N=99)
Provide consultation/facilitation	6	21	15	3	1	10	5	1	5	7	74
Conduct educational meetings	4	14	6	3	0	7	3	4	5	7	53
Distribute educational materials	3	9	5	0	1	8	5	3	2	3	39
Conduct ongoing training	2	1	7	1	1	3	0	0	3	2	20
Conduct educational outreach visits	3	3	5	0	0	0	1	0	2	3	17
Make training dynamic	1	3	2	0	3	3	0	1	2	2	17
Develop educational materials	0	1	3	1	0	5	2	0	2	3	17
Create a learning collaborative	0	0	6	0	0	2	2	0	0	2	12
Audit and provide feedback	5	11	14	1	1	2	2	2	3	3	44
Develop formal impl. blueprint	1	4	8	1	0	1	1	1	2	6	25
Conduct local needs assessment	1	5	5	1	1	5	1	0	1	1	21
Assess for readiness and identify barriers and facilitators	0	2	6	0	1	2	2	0	2	3	18
Promote network weaving	0	1	6	2	1	5	4	0	1	2	22
Model and simulate change	3	6	3	1	0	3	0	0	0	1	17
Identify and prepare champions	0	2	5	2	0	1	0	1	3	1	15

Org. clinician impl. team meetings	0	2	7	2	0	0	0	0	1	1	13
Tailor strategies	4	7	7	0	2	6	3	1	2	5	37
Promote adaptability	2	2	7	3	0	1	0	1	2	1	19

Table ERA6: Overview of Included RCTs (N=30)

					Outcomes			
**Study**	**Aim**	**Method**	**ISP role**	**ISP strategies reported**	***Individual / Organization***	***Implementation***	***Service***	***Client***
**Acolet et al. (2011)**	To assess whether an active strategy for the dissemination of neonatal research findings, recommendations and guidelines was more likely to change policy and practice than the passive dissemination of a report, a slide package and a position statement.	Cluster RCT	Other (regional champion)	Audit & feedback; champions; dynamic training; implementation blueprint [N=4]	Opportunity: non-significant	N/A[1]	Timeliness / Safety: mixed	N/A
**Beidas et al., 2012**	To evaluate the efficacy of three different brief CBT training modalities, and to evaluate the impact of including ongoing consultation after this training on therapist CBT knowledge, skill and adherence and training satisfaction.	RCT	Consultant	Consultation/ facilitation; develop educational materials; dynamic training; educational meetings [N=4]	Capacity: mixed	Fidelity: non-significant; Adherence: significant	N/A	N/A
**Bradshaw et al., 2012**	To compare student outcomes achieved by PBIS schools that receive (a) traditional training and support in tier 1 implementation from the state and district, and (b) tier 2 training and coaching	Cluster RCT	Coach	Consultation/ facilitation; distribute educational materials; educational meetings; ongoing training [N=4]	Capacity: significant	Fidelity: non-significant	Efficiency: significant	Student achievement: Significant

[1] N/A = not applicable

	from an external tier 2 coach (i.e. PBISplus = intervention condition) with those from PBIS schools that receive traditional training and support in tier 1 implementation from the state and district only (i.e. SWPBIS = comparison/ control condition).							
**Brown et al., 2014**	To determine whether participation in the Community Development Team (CDT) model improves the rates of program adoption, implementation, and fidelity of Multidimensional Treatment Foster Care (MTFC) in 51 counties (56 sites) in California and Ohio when compared with an individualized implementation condition (IND).	Cluster RCT	Facilitator	Assess readiness; consultation/ facilitation; educational meetings; learning collaborative [N=4]	N/A	Adoption: non-significant; fidelity: non-significant; process: non-significant	N/A	N/A
**Calo et al., 2018**	To compare the effectiveness of quality improvement coaching when delivered either in-person or based on webinars on improving HPV vaccination coverage.	Cluster RCT	Coach	Audit & feedback; distribute educational materials; educational meetings; implementation blueprint; [N=4]	Capacity: non-significant	Adoption: non-significant; feasibility: non-significant; acceptability: non-significant; costs: mixed	N/A	N/A

**Chinman et al., 2017**	To evaluate how much a Getting to Outcomes (GTO) guided implementation strategy (training + manuals + implementation support) facilitated the adoption and implementation of the MISSION-Vet intervention targeting (formerly) homeless veterans – when compared with implementation as usual (training + manuals only).	Cluster RCT	TA provider	Audit & feedback; consultation/ facilitation; develop educational materials; distribute educational materials; educational outreach; implementation blueprint; organize meetings; promote adaptability; tailor strategies [N=9]	N/A	Adoption: significant; penetration: significant	N/A	N/A
**Chinman et al., 2018**	To assess the effectiveness of the GTO Implementation Support Intervention by comparing the outcomes from 15 Boys and Girls Club sites implementing CHOICE (control group), a five-session evidence-based alcohol and drug prevention program, with 14 similar sites implementing CHOICE supported by GTO (intervention group).	Cluster RCT	TA provider	Audit & feedback; educational meetings; implementation blueprint; ongoing training [N=4]	N/A	Fidelity: significant	N/A	Functioning: non-significant
**Chung et al., 2015**	To examine whether the implementation approach CEP (Community and Engagement Planning) is more effective in (a) generating options for	Cluster RCT	Unlabeled	Educational meetings; educational outreach [N=2]	Opportunity (training participation): Significant	N/A	N/A	N/A

	training depression care quality improvement (DCQI), and (b) increasing mean hours practitioners spend on DCQI training than the implementation approach RS (Resources for Services).							
**Dickinson et al., 2014**	To compare the influence of three approaches for implementing the Chronic Care Model on changing the practice culture of primary care clinics and improving the quality of their diabetes care: (1) Practice facilitation over 6 months using a reflective adaptive process (RAP); (2) Practice facilitation for up to 18 months using continuous quality improvement (CQI); and (3) Self-directed practice, without facilitation (SD).	Cluster RCT	Facilitator	Audit & feedback; organize meetings [N=2]	Opportunity (culture): Mixed	Adoption: significant (CQI compared with RAP and SD)	N/A	N/A
**Eiraldi et al., 2018**	To test which of two consultation strategies (Consultation versus Consultation+) used with school counselors resulted in (a) higher treatment content and process fidelity of an intervention aimed at externalizing behaviors in school	Cluster RCT	Consultant	Audit & feedback; consultation/ facilitation; distribute educational materials; dynamic training; educational meetings; model change; tailor strategies [N=7]	N/A	Fidelity: significant	N/A	Wellbeing: non-significant

	children (CPP); and (b) better child outcomes (changes in diagnostic status and impairment).							
**Funderburk et al., 2015**	To answer the following research question: Does live video consultation improve downstream client outcomes relative to standard phone consultation?	RCT	Consultant	Consultation/ facilitation; model change [N=2]	N/A	N/A	N/A	Functioning: Significant
**Glisson et al., 2013**	To assess (a) whether the Availability, Responsiveness and Continuity (ARC) organizational intervention improved youth outcomes in community based mental health programs and (b) whether programs with more improved organizational social contexts following the 18-month ARC intervention had better youth outcomes than programs with less improved social contexts.	Cluster RCT	Other (ARC specialist)	Assess readiness; distribute educational materials; educational outreach [N=3]	N/A	N/A	N/A	Wellbeing: Significant
**Gustafson et al., 2013**	To test the effectiveness of four different quality improvement interventions to reduce patients' waiting time, retention and to increase the number of patients served annually	Cluster RCT	Mix (QI experts + coaches)	Consultation/ facilitation; distribute educational materials; educational outreach; implementation	N/A	Penetration: significant	Timeliness: significant; client/patient centeredness: non-significant	N/A

	across addiction clinics in the U.S.			blueprint; learning collaborative; tailor strategies [N=6]				
**Kelly et al., 2000**	To compare the effectiveness of 3 dissemination strategies for transferring HIV prevention models from the research arena to community providers of HIV prevention services.	Cluster RCT	Consultant	Consultation/ facilitation; distribute educational materials; educational meetings; tailor strategies [N=4]	N/A	Adoption: significant	N/A	N/A
**Kinley et al., 2014**	To examine the impact of providing high facilitation (HF) when implementing the Gold Standard Framework for Care Homes (GSFCH) program	Cluster RCT	Facilitator	Educational meetings; model change; ongoing training [N=3]	N/A	Adoption: mixed	Efficiency: significant	Wellbeing: non-significant
**Leathers et al., 2016**	To investigate the effect of post-training change agent interactions on the use of an evidence-based intervention relative to training as usual with no additional post-training support.	RCT	Consultant	Consultation/ facilitation; dynamic training; educational meetings; distribute educational materials [N=4]	N/A	Adoption: non-significant	N/A	N/A
**Lemelin et al., 2011**	To test the effectiveness of a tailored multifaceted facilitation intervention to improve preventive care in capitation-based family practices	Cluster RCT	Facilitator	Audit & feedback; develop educational materials; implementation blueprint; promote adaptability; tailor strategies [N=5]	N/A	Adoption: significant	Efficiency: significant	N/A
**Meropol et al., 2014**	To assess the effectiveness of practice facilitation, i.e. practice-tailored rapid-	Cluster RCT	Facilitator	Audit & feedback; consultation/ facilitation;	N/A	N/A	Efficiency: significant	N/A

	cycle feedback/change strategies, on improving obesity screening/ counseling, lead screening, and dental fluoride varnish application in pediatric preventive services.			distribute educational materials; implementation blueprint; ongoing training; promote adaptability; tailor strategies [N=7]				
**Mold et al., 2008**	To assess the effectiveness of a multicomponent implementation strategy aimed at increasing the use of three processes (standing orders – reminder/recall systems – wellness visits) shown to have increased the delivery of preventive services through primary practices; to identify contextual barriers to the implementation of these processes; and to measure the impact of these processes on rates of delivery of preventive services	RCT	Facilitator	Audit & feedback; consultation/ facilitation; distribute educational materials; educational outreach [N=4]	N/A	Adoption: significant	Efficiency: mixed	N/A
**Mold et al., 2014**	To compare the independent and combined effectiveness of Practice facilitation (PF) and local learning collaboratives (LLCs), combined with performance feedback and academic detailing, with	Cluster RCT	Facilitator	Distribute educational materials; implementation blueprint; learning collaborative [N=3]	N/A	Adoption: significant	N/A	N/A

	performance feedback and academic detailing alone on the implementation of the National Heart, Lung and Blood Institute's Asthma Guidelines.							
**Murray et al., 2018**	To examine whether the initial model of Together facing the Challenge (TFTC) could be improved to increase sustainability and improve implementation	Cluster RCT	Consultant	Consultation/ facilitation; distribute educational materials; educational meetings [N=3]	Opportunity: significant	Fidelity: non-significant	Efficiency: non-significant	N/A
**Palinkas et al., 2017**	To determine whether use of research evidence (URE) was independently associated with the stage of implementation of Treatment Foster Care Oregon (TFC-O) and the proportion of activities completed at the pre-implementation, implementation and sustainment phases; and whether URE was significantly associated with the QIC strategy used to scale up the EBP – the Community Development Team (CDT)	Cluster RCT	Consultant	Assess readiness; consultation/ facilitation; educational meetings; tailor strategies [N=4]	N/A	Adoption: mixed	N/A	N/A
**Parchman et al., 2013**	To assess the effectiveness of practice facilitation to improve the organization and delivery of diabetes	Cluster RCT	Facilitator	Audit & feedback; organize meetings; promote adaptability [N=3]	N/A	Adoption: significant	N/A	N/A

	care in small, autonomous primary care practices.							
**Peterson et al., 2015**	To assess whether a prevention support system (i.e., “enhanced support system”) that builds capacity through the provision of technical assistance, best practice guides, and direct consultation can successfully bridge the gap between fall prevention research and the implementation of an evidence-driven, community-based fall prevention program.	Cluster RCT	Mix (Injury Research Centre faculty staff and study team)	Assess readiness; audit & feedback; consultation/ facilitation; distribute educational materials; educational meetings; needs assessment; promote network weaving [N=7]	N/A	N/A	N/A	Functioning: significant
**Quanbeck et al., 2018**	To pilot test the blended implementation strategy ‘systems consultation’ (SC) in a small set of primary care clinics to see if the strategy demonstrated feasibility, acceptability, and preliminary effectiveness in improving clinician adherence to opioid-prescribing guidelines and reducing morphine-equivalent daily dose (MEDD) for patients on long-term opioid therapy	RCT	Consultant	Audit & feedback; champions; consultation/ facilitation; educational meetings; educational outreach; needs assessment; organize meetings; promote adaptability; tailor strategies [N=9]	N/A	N/A	Efficiency: Mixed	N/A

**Rohrbach et al., 2010**	To test the effectiveness of a comprehensive training condition to produce stronger implementation fidelity and immediate treatment outcomes	RCT	Unlabeled	Consultation/ facilitation; educational meetings; distribute educational meetings [N=3]	Capacity: significant	Acceptability: significant, fidelity: significant	N/A	Functioning: significant
**Seers et al., 2018**	To extend knowledge of facilitation as a process for getting research evidence into practice by testing the effectiveness of and evaluating the contribution that two different models of facilitation can make to implementing evidence-based urinary continence recommendations into practice.	Cluster RCT	Facilitator	Audit & feedback; consultation/ facilitation; develop educational materials; distribute educational materials; educational meetings; implementation blueprint [N=6]	N/A	Adoption: non-significant	N/A	N/A
**L. Williams et al., 2016**	To assess the effectiveness of quality improvement training plus indicator feedback on deep vein thrombosis (DVT) prophylaxis and dysphagia (swallowing difficulties) screening when compared with indicator feedback alone	Cluster RCT	Coaches in pairs (systems engineer + implementation researcher)	Audit & feedback; consultation/ facilitation; educational meetings; educational outreach [N=4]	N/A	N/A	Efficiency: non-significant	N/A
**N. Williams et al., 2017**	To examine the cross-level contextual, motivational, and barrier-related mechanisms that explain the effect of ARC (Availability, Responsiveness, and	Cluster RCT	Other (ARC specialist)	Consultation/ facilitation; distribute educational materials; educational outreach [N=3]	N/A	Adoption: significant; feasibility: significant	N/A	N/A

	Continuity implementation support strategy) on clinicians' EBP adoption and use							
**Yano et al., 2008**	To evaluate the impact of a locally adapted evidence-based quality improvement (EBQI) approach to the implementation of smoking cessation guidelines into routine practice of VA primary care practices	Cluster RCT	Unlabeled	Audit & feedback; champions; consultation/ facilitation; distribute educational materials; educational meetings; implementation blueprint; promote adaptability [N=7]	N/A	Penetration: Significant	N/A	Functioning: non-significant

Table ERA7a: Quality Assessment Questions used with RCTs

Consideration	Questions	Responses
**Appropriate implementation intervention development**	(1) Has the design of the implementation intervention been informed by appropriate theory or an implementation framework?	Yes / No<br>Please name the theory / framework
	(2) Has the design of the implementation intervention been informed by local empirical evidence?	Yes / No<br>Please describe the empirical evidence
**Categorization of implementation strategies**	(3) Has a taxonomy been used in describing implementation strategies used in the study, e.g. the ERIC compilation (Powell, Waltz et al., 2015) or the Effective Practice and Organization of Care (EPOC, 2015) taxonomy?	Yes / No<br>Please name the taxonomy
**Appropriate outcomes for implementation research**	(4) Which outcomes have been reported for this study?	(1) IMPLEMENTATION OUTCOMES: Acceptability; Adoption; Appropriateness; Costs; Feasibility; Fidelity; Penetration; Sustainability (multiple responses possible)<br>(2) SERVICE OUTCOMES<br>(3) CLIENT OUTCOMES
	(5) Have adverse effects of the implementation intervention been considered?	Yes / No

Table ERA7b: Quality Assessment of Included RCTs (N=30)

				**OUTCOMES ASSESSED**		
**STUDY**	**FRAMEWORK/THEORY USE**	**USE OF EMPIRICAL EVIDENCE**	**STRATEGY TAXONOMY USE**	***Implementation***	***Service***	***Client***
**Acolet (2011)**	NO	From study protocol: insufficient data from a survey assessing the impact of a dissemination package to improve knowledge transfer in the area of care of premature babies in England		NO	Timeliness<br>Safety	NO
**Beidas (2012)**	NO	NO	NO	Fidelity	NO	NO

**Bradshaw (2012)**	PBIS*plus* theory of change model (p.189).			Fidelity	Efficiency	Functioning
**Calo (2018)**	NO	Previous experience with using the AFIX coaching model that is in focus of this study plus input from health specialists from three different states	NO	Adoption<br>Feasibility<br>Acceptability<br>Costs	NO	NO
**Chinman (2017)**	Getting to Outcomes + RE-AIM + Consolidated Framework for Implementation Research	NO	NO	Adoption<br>Penetration		
**Chinman (2018)**	Getting to Outcomes	NO	NO	Fidelity	NO	Functioning
**Chung (2015)**	NO	NO	NO	NO	NO	NO
**Dickinson (2014)**	Continuous Quality Improvement theory and reflective adaptive process theory.	NO	NO	Adoption	NO	NO
**Eiraldi (2018)**	NO	NO	NO	Fidelity	NO	Wellbeing
**Funderburk (2015)**	NO	NO	NO	NO	NO	Functioning
**Glisson (2013)**	Organizational culture theory	NO	NO	NO	NO	Functioning
**Gustafson (2013)**	NO	NO	NO	Penetration	Timeliness<br>Client-Centeredness	NO
**Hendricks Brown (2014)**	NO	NO	NO	Adoption<br>Fidelity<br>Sustainability<br>Process (SIC)	NO	NO
**Kelly (2000)**	NO	NO	NO	Adoption	NO	NO
**Kinley (2014)**	A single and very brief reference to the PARiHS used to explain why high facilitation strategy was used.	NO	NO	Adoption	Efficiency	Wellbeing
**Leathers (2016)**	Rogers diffusion of innovations theory applied in detail (pp. 4/5)	NO	NO	Adoption	NO	NO
**Lemelin (2001)**	Refers to a seven-item CQI framework developed by Leininger et al. as guiding facilitation.	NO	NO	Adoption	Efficiency	NO

**Meropol (2014)**	NO	NO	NO	NO	Efficiency	NO
**Mold (2008)**	NO	NO	NO	Adoption	NO	NO
**Mold (2014)**	NO	NO	NO	Adoption	NO	NO
**Murray (2018)**	NO	NO	NO	Fidelity	NO	Wellbeing
**Palinkas (2017)**	NO	NO	NO	Adoption	NO	NO
**Parchman (2013)**	NO. On page 6, the authors highlight the use of theory for intervention development as a strength of the study, but from the remaining text it was unclear what theory might have been used.	NO	NO	Adoption	NO	NO
**Peterson (2015)**	Interactive Systems Framework + RE-AIM: detailed use with ISF informing study frame and RE-AIM organizing use of measures	NO	NO	NO	NO	Functioning
**Quanbeck (2018)**	RE-AIM was used to structure outcome measurement	Implementation support strategies developed in collaboration developers of clinical guideline to be implemented locally.	ERIC	Acceptability<br>Feasibility	Efficiency	NO
**Rohrbach (2010)**	NO	NO	NO	Acceptability<br>Fidelity	NO	Functioning
**Seers (2018)**	Theories underpinning the two facilitation approaches used in this study, including the PARiHS, have been briefly described in a supplementary file: CQI theory, Theory on org. learning & critical social sciences.	NO	NO	Adoption	NO	NO
**Williams, Linda (2016)**	NO	NO	NO	NO	Efficiency	NO
**Williams, N. (2016)**	Org. culture theory and the theory of planned behavior. Also uses Proctor's implementation outcome framework	NO	NO	Adoption<br>Feasibility	NO	NO
**Yano (2008)**	NO	NO	NO	Penetration	NO	Functioning

Included Publications (N=109)

Aasekjær, K., Waehle, H. V., Ciliska, D., Nordtvedt, M. W., & Hjälmhult, E. (2016). Management Involvement - A Decisive Condition When Implementing Evidence-Based Practice. *Worldviews on Evidence-Based Nursing*, *13*(1), 32–41. https://dx.doi.org/10.1111/wvn.12141

Acolet, D., Allen, E., Houston, R., Wilkinson, A. R., Costeloe, K., & Elbourne, D. (2011). Improvement in Neonatal Intensive Care Unit Care: A Cluster Randomised Controlled Trial of Active Dissemination of Information. *Archives of Disease in Childhood - Fetal and Neonatal Edition*, *96*(6), F434-F439. https://dx.doi.org/10.1136/adc.2010.207522

Akin, B. A. (2016). Practitioner Views on the Core Functions of Coaching in the Implementation of an Evidence-Based Intervention in Child Welfare. *Children and Youth Services Review*, *68*, 159–168. https://dx.doi.org/10.1016/j.childyouth.2016.07.010

Anaby, D., Korner-Bitensky, N., Law, M., & Cormier, I. (2014). Focus on Participation for Children and Youth with Disabilities: Supporting Therapy Practice Through a Guided Knowledge Translation Process. *British Journal of Occupational Therapy*, *78*(7), 440–449. https://dx.doi.org/10.1177/0308022614563942

Anyon, Y., Nicotera, N., & Veeh, C. A. (2016). Contextual Influences on the Implementation of a Schoolwide Intervention to Promote Students' Social, Emotional, and Academic Learning. *Children & Schools*, *38*(2), 81–88. https://dx.doi.org/10.1093/cs/cdw008

Barac, R., Kimber, M., Johnson, S., & Barwick, M. (2018). The Effectiveness of Consultation for Clinicians Learning to Deliver Motivational Interviewing with Fidelity. *Journal of Evidence-Informed Social Work*, *15*(5), 1–24. https://dx.doi.org/10.1080/23761407.2018.1480988

Becker, K. D., Bradshaw, C. P., Domitrovich, C., & Ialongo, N. S. (2013). Coaching Teachers to Improve Implementation of the Good Behavior Game. *Administration and Policy in Mental Health and Mental Health Services Research*, *40*(6), 482–493. https://dx.doi.org/10.1007/s10488-013-0482-8

Beidas, R. S., Edmunds, J. M., Marcus, S. C., & Kendall, P. C. (2012). Training and Consultation to Promote Implementation of an Empirically Supported Treatment: A Randomized Trial. *Psychiatric Services*, *63*(7), 660–665. https://dx.doi.org/10.1176/appi.ps.201100401

Beidas, R. S., Edmunds, J. M., Cannuscio, C. C., Gallagher, M., Downey, M. M., & Kendall, P. C. (2013). Therapists Perspectives on the Effective Elements of Consultation Following Training. *Administration and Policy in Mental Health and Mental Health Services Research*, *40*(6), 507–517. https://dx.doi.org/10.1007/s10488-013-0475-7

Bice-Urbach, B. J., & Kratochwill, T. R. (2016). Teleconsultation: The Use of Technology to Improve Evidence-Based Practices in Rural Communities. *Journal of School Psychology*, *56*, 27–43. https://dx.doi.org/10.1016/j.jsp.2016.02.001

Bidassie, B., Williams, L. S., Woodward-Hagg, H., Matthias, M. S., & Damush, T. M. (2015). Key Components of External Facilitation in an Acute Stroke Quality Improvement Collaborative in the Veterans Health Administration. *Implementation Science*, *10*, Article 69. https://dx.doi.org/10.1186/s13012-015-0252-y

Bradshaw, C. P., Pas, E. T., Goldweber, A., Rosenberg, M. S., & Leaf, P. J. (2012). Integrating School-Wide Positive Behavioral Interventions and Supports with Tier 2 Coaching to Student Support Teams: The PBISplus model. *Advances in School Mental Health Promotion*, *5*(3), 177–193. https://dx.doi.org/10.1080/1754730x.2012.707429

Brown, C.H., Chamberlain, P., Saldana, L., Padgett, C., Wang, W., & Cruden, G. (2014). Evaluation of two Implementation Strategies in 51 Child County Public Service Systems in Two States: Results of a Cluster Randomized Head-to-Head Implementation Trial. *Implementation Science*, *9,* Article 134. https://dx.doi.org/10.1186/s13012-014-0134-8

Brown, K. M., Elliott, S. J., Robertson-Wilson, J., Vine, M. M., & Leatherdale, S. T. (2018). "Now What?" Perceived Factors Influencing Knowledge Exchange in School Health Research. *Health Promotion Practice*, *19*(4), 590–600. https://dx.doi.org/10.1177/1524839917732037

Brown, K. M., Elliott, S. J., & Leatherdale, S. T. (2018). Researchers Supporting Schools to Improve Health: Influential Factors and Outcomes of Knowledge Brokering in the COMPASS Study. *Journal of School Health*, *88*(1), 54–64. https://dx.doi.org/10.1111/josh.12578

Brown, K. M., Elliott, S. J., Robertson-Wilson, J., Vine, M. M., & Leatherdale, S. T. (2018). Can Knowledge Exchange Support the Implementation of a Health-Promoting Schools Approach? Perceived Outcomes of Knowledge Exchange in the COMPASS study. *BMC Public Health*, *18(*Article 351). https://dx.doi.org/10.1186/s12889-018-5229-8

Brownson, R. C., Ballew, P., Brown, K. L., Elliott, M. B., Haire-Joshu, D., Heath, G. W., & Kreuter, M. W. (2007). The Effect of Disseminating Evidence-Based Interventions That Promote Physical Activity to Health Departments. *American Journal of Public Health*, *97*(10), 1900–1907. https://dx.doi.org/10.2105/ajph.2006.090399

Brunette, M. F., Asher, D., Whitley, R., Lutz, W. J., Wieder, B. L., Jones, A. M., & McHugo, G. J. (2008). Implementation of Integrated Dual Disorders Treatment: A Qualitative Analysis of Facilitators and Barriers. *Psychiatric Services*, *69*(9), 989–995. https://dx.doi.org/10.1176/ps.2008.59.9.989

Burns, M. K., Peters, R., & Noell, G. H. (2008). Using Performance Feedback to Enhance Implementation Fidelity of the Problem-Solving Team Process. *Journal of School Psychology*, *46*(5), 537–550. https://dx.doi.org/10.1016/j.jsp.2008.04.001

Byrnes, A., Young, A., Mudge, A., Banks, M., Clark, D., & Bauer, J. (2018). Prospective Application of an Implementation Framework to Improve Postoperative Nutrition Care Processes: Evaluation of a Mixed Methods Implementation Study. *Nutrition & Dietetics*, *75*(4), 353–362. https://dx.doi.org/10.1111/1747-0080.12464

Calo, W. A., Gilkey, M. B., Leeman, J., Heisler-MacKinnon, J., Averette, C., Sanchez, S., … Brewer, N. T. (2018). Coaching Primary Care Clinics for HPV Vaccination Quality Improvement: Comparing In-Person and Webinar Implementation. *Translational Behavioral Medicine*, *9*(1), 23–31. https://dx.doi.org/10.1093/tbm/iby008

Cameron, D., Russell, D. J., Rivard, L., Darrah, J., & Palisano, R. (2011). Knowledge Brokering in Children's Rehabilitation Organizations: Perspectives from Administrators. *Journal of Continuing Education in the Health Professions*, *31*(1), 28–33. https://dx.doi.org/10.1002/chp.20098

Caron, E., & Dozier, M. (2019). Effects of Fidelity-Focused Consultation on Clinicians' Implementation: An Exploratory Multiple Baseline Design. *Administration and Policy in Mental Health and Mental Health Services Research*, *46*(4), 445–457. doi: https://dx.doi.org/10.1007/s10488-019-00924-3

Carson, R. L., Castelli, D. M., Kuhn, A. C. P., Moore, J. B., Beets, M. W., Beighle, A., … Glowacki, E. M. (2014). Impact of Trained Champions of Comprehensive School Physical Activity

Programs on School Physical Activity Offerings, Youth Physical Activity and Sedentary Behaviors. *Preventive Medicine*, *69*, S12–S19. https://dx.doi.org/10.1016/j.ypmed.2014.08.025

Chaffin, M. J., Hecht, D., Aarons, G. A., Fettes, D. L., Hurlburt, M., & Ledesma, K. (2015). EBT Fidelity Trajectories Across Training Cohorts Using the Interagency Collaborative Team Strategy. *Administration and Policy in Mental Health and Mental Health Services Research*, *43*(2), 144–156. https://dx.doi.org/10.1007/s10488-015-0627-z

Chaple, M., & Sacks, S. (2016). The Impact of Technical Assistance and Implementation Support on Program Capacity to Deliver Integrated Services. *The Journal of Behavioral Health Services & Research*, *43*(1), 3–17. https://dx.doi.org/10.1007/s11414-014-9419-6

Cheron, D. M., Chiu, A. A. W., Stanick, C. F., Stern, H. G., Donaldson, A. R., Daleiden, E. L., & Chorpita, B. F. (2019). Implementing Evidence Based Practices for Children's Mental Health: A Case Study in Implementing Modular Treatments in Community Mental Health. *Administration and Policy in Mental Health and Mental Health Services Research*, *46*(3), 391–410. https://dx.doi.org/10.1007/s10488-019-00922-5

Chew, S., Armstrong, N., & Martin, G. (2013). Institutionalising Knowledge Brokering as a Sustainable Knowledge Translation Solution in Healthcare: How Can it Work in Practice? *Evidence & Policy: A Journal of Research, Debate and Practice*, *9*(3), 335–351. https://dx.doi.org/10.1332/174426413x662734

Chilenski, S. M., Perkins, D. F., Olson, J., Hoffman, L., Feinberg, M. E., Greenberg, M., … Spoth, R. (2016). The Power of a Collaborative Relationship Between Technical Assistance Providers and Community Prevention Teams: A Correlational and Longitudinal Study. *Evaluation and Program Planning*, *54*, 19–29. https://dx.doi.org/10.1016/j.evalprogplan.2015.10.002

Chinman, M. J., McCarthy, S., Hannah, G., Byrne, T. H., & Smelson, D. A. (2017). Using Getting To Outcomes to Facilitate the Use of an Evidence-Based Practice in VA Homeless Programs: A Cluster-Randomized Trial of an Implementation Support Strategy. *Implementation Science*, *12*, Article 34. https://dx.doi.org/10.1186/s13012-017-0565-0

Chinman, M. J., Ebener, P., Malone, P. S., Cannon, J., D'Amico, E. J., & Acosta, J. (2018). Testing Implementation Support for Evidence-Based Programs in Community Settings: A Replication Cluster-Randomized Trial of Getting To Outcomes®. *Implementation Science*, *13*, Article 131. https://dx.doi.org/10.1186/s13012-018-0825-7

Chung, B., Ngo, V. K., Ong, M. K., Pulido, E., Jones, F., Gilmore, J., … Miranda, J. (2015). Participation in Training for Depression Care Quality Improvement: A Randomized Trial of Community Engagement or Technical Support. *Psychiatric Services*, *66*(8), 831–839. https://dx.doi.org/10.1176/appi.ps.201400099

Dickinson, W. P., Dickinson, L. M., Nutting, P. A., Emsermann, C. B., Tutt, B., Crabtree, B. F., … West, D. R. (2014). Practice Facilitation to Improve Diabetes Care in Primary Care: A Report from the EPIC Randomized Clinical Trial. *The Annals of Family Medicine*, *12*(1), 8–16. https://dx.doi.org/10.1370/afm.1591

Dobbins, M., Traynor, R. L., Workentine, S., Yousefi-Nooraie, R., & Yost, J. (2018). Impact of an Organization-Wide Knowledge Translation Strategy to Support Evidence-Informed Public Health Decision Making. *BMC Public Health*, *18*, Article 1412. https://dx.doi.org/10.1186/s12889-018-6317-5

Dogherty, E. J., Harrison, M. B., Graham, I. D., Vandyk, A. D., & Keeping-Burke, L. (2013). Turning Knowledge into Action at the Point-of-Care: The Collective Experience of Nurses Facilitating

the Implementation of Evidence-Based Practice. *Worldviews on Evidence-Based Nursing*, *10*(3), 129–139. https://dx.doi.org/10.1111/wvn.12009

Dogherty, E. J., Harrison, M. B., Baker, C., & Graham, I. D. (2012). Following a Natural Experiment of Guideline Adaptation and Early Implementation: A Mixed-Methods Study of Facilitation. *Implementation Science*, *7*(Article 9). https://dx.doi.org/10.1186/1748-5908-7-9

Duffy, J. L., Prince, M. S., Johnson, E. E., Alton, F. L., Flynn, S., Faye, A. M., … Hinzey, A. L. (2012). Enhancing Teen Pregnancy Prevention in Local Communities: Capacity Building Using the Interactive Systems Framework. *American Journal of Community Psychology*, *50*(3–4), 370–385. https://dx.doi.org/10.1007/s10464-012-9531-9

Dusenbury, L., Hansen, W. B., Jackson-Newsom, J., Pittman, D. S., Wilson, C. V., Nelson-Simley, K., … Giles, S. M. (2010). Coaching to Enhance Quality of Implementation in Prevention. *Health Education*, *110*(1), 43–60. https://dx.doi.org/10.1108/09654281011008744

Eiraldi, R., Mautone, J. A., Khanna, M. S., Power, T. J., Orapallo, A., Cacia, J., … Jawad, A. F. (2018). Group CBT for Externalizing Disorders in Urban Schools: Effect of Training Strategy on Treatment Fidelity and Child Outcomes. *Behavior Therapy*, *49*, 538–550. https://dx.doi.org/10.1016/j.beth.2018.01.001.

Elnitsky, C. A., Powell-Cope, G., Besterman-Dahan, K. L., Rugs, D., & Ullrich, P. M. (2015). Implementation of Safe Patient Handling in the U.S. Veterans Health System: A Qualitative Study of Internal Facilitators' Perceptions. *Worldviews on Evidence-Based Nursing*, *12*(4), 208–216. https://dx.doi.org/10.1186/1748-5908-6-99

Feinberg, M. E., Ridenour, T. A., & Greenberg, M. T. (2008). The Longitudinal Effect of Technical Assistance Dosage on the Functioning of Communities That Care Prevention Boards in Pennsylvania. *The Journal of Primary Prevention*, *29*(2), 145–165. https://dx.doi.org/10.1007/s10935-008-0130-3

Fortney, J. C., Pyne, J. M., Ward-Jones, S., Bennett, I. M., Diehl, J., Farris, K., … Curran, G. M. (2018). Implementation of Evidence-Based Practices for Complex Mood Disorders in Primary Care Safety Net Clinics. *Families, Systems, & Health*, *36*(3), 267–280. https://dx.doi.org/10.1037/fsh0000357

Franks, R. P., & Bory, C. T. (2015). Who Supports the Successful Implementation and Sustainability of Evidence-Based Practices? Defining and Understanding the Roles of Intermediary and Purveyor Organizations. *New Directions for Child and Adolescent Development*, *2015*(149), 41–56. https://dx.doi.org/10.1002/cad.20112

Funderburk, B., Chaffin, M., Bard, E., Shanley, J., Bard, D., & Berliner, L. (2014). Comparing Client Outcomes for Two Evidence-Based Treatment Consultation Strategies. *Journal of Clinical Child & Adolescent Psychology*, *44*(5), 730–741. https://dx.doi.org/10.1080/15374416.2014.910790

Garbacz, S. A., Watkins, N. D., Diaz, Y., Jr., E. R. B., Schwartz, B., & Eiraldi, R. (2016). Using Conjoint Behavioral Consultation to Implement Evidence-Based Practices for Students in Low-Income Urban Schools. *Preventing School Failure: Alternative Education for Children and Youth*, *61*(3), 1–13. https://dx.doi.org/10.1080/1045988x.2016.1261078

Gerrish, K., McDonnell, A., Nolan, M., Guillaume, L., Kirshbaum, M., & Tod, A. (2011). The Role of Advanced Practice Nurses in Knowledge Brokering as a Means of Promoting Evidence-Based Practice Among Clinical Nurses. *Journal of Advanced Nursing*, *67*(9), 2004–2014. https://dx.doi.org/10.1111/j.1365-2648.2011.05642.x

Gerrish, K., Nolan, M., McDonnell, A., Tod, A., Kirshbaum, M., & Guillaume, L. (2012). Factors Influencing Advanced Practice Nurses' Ability to Promote Evidence-Based Practice among Frontline Nurses. *Worldviews on Evidence-Based Nursing*, *9*(1), 30–39. https://dx.doi.org/10.1111/j.1741-6787.2011.00230.x

Glisson, C., Hemmelgarn, A., Green, P., & Williams, N. J. (2013). Randomized Trial of the Availability, Responsiveness and Continuity (ARC) Organizational Intervention for Improving Youth Outcomes in Community Mental Health Programs. *Journal of the American Academy of Child & Adolescent Psychiatry*, *52*(5), 493–500. https://dx.doi.org/10.1016/j.jaac.2013.02.005

Graaf, G., McBeath, B., Lwin, K., Holmes, D., & Austin, M. J. (2016). Supporting Evidence-Informed Practice in Human Service Organizations: An Exploratory Study of Link Officers. *Human Service Organizations: Management, Leadership & Governance*, *41*(1), 1–18. https://dx.doi.org/10.1080/23303131.2016.1192575

Gunderson, L. M., Willging, C. E., Jaramillo, E. M. T., Green, A. E., Fettes, D. L., Hecht, D. B., & Aarons, G. A. (2018). The Good Coach: Implementation and Sustainment Factors That Affect Coaching as Evidence-Based Intervention Fidelity Support. *Journal of Children's Services*, *Volume 13*(1), 1–17. https://dx.doi.org/10.1108/jcs-09-2017-0043

Gustafson, D. H., Quanbeck, A. R., Robinson, J. M., Ford, J. H., Pulvermacher, A., French, M. T., … McCarty, D. (2013). Which Elements of Improvement Collaboratives are Most Effective? A Cluster-Randomized Trial. *Addiction*, *108*(6), 1145–1157. https://dx.doi.org/10.1111/add.12117

Hodge, L. M., Turner, K. M. T., Sanders, M. R., & Forster, M. (2017). Factors That Influence Evidence-Based Program Sustainment for Family Support Providers in Child Protection Services in Disadvantaged Communities. *Child Abuse & Neglect*, *70*, 134–145. https://dx.doi.org/10.1016/j.chiabu.2017.05.017

Holtrop, J. S., Baumann, J., Arnold, A. K., & Torres, T. (2008). Nurses as Practice Change Facilitators for Healthy Behaviors. *Journal of Nursing Care Quality*, *23*(2), 123–131. https://dx.doi.org/10.1097/01.ncq.0000313761.79396.37

Hurlburt, M., Aarons, G. A., Fettes, D. L., Willging, C. E., Gunderson, L. M., & Chaffin, M. J. (2014). Interagency Collaborative Team Model for Capacity Building to Scale-Up Evidence-Based Practice. *Children and Youth Services Review*, *39*(C), 160–168. https://dx.doi.org/10.1016/j.childyouth.2013.10.005

Hurtubise, K., Rivard, L., Héguy, L., Berbari, J., & Camden, C. (2016). Virtual Knowledge Brokering: Describing the Roles and Strategies Used by Knowledge Brokers in a Pediatric Physiotherapy Virtual Community of Practice. *Journal of Continuing Education in the Health Professions*, *36*(3), 186–194. https://dx.doi.org/10.1097/ceh.0000000000000101

Jacobson, N., Johnson, R., Deyo, B., Alagoz, E., & Quanbeck, A. (2019). Systems Consultation for Opioid Prescribing in Primary Care: A Qualitative Study of Adaptation. *BMJ Quality & Safety*, *28*(5), 397–404. https://dx.doi.org/10.1136/bmjqs-2018-008160

Kaasalainen, S., Ploeg, J., Donald, F., Coker, E., Brazil, K., Martin-Misener, R., … Hadjistavropoulos, T. (2015). Positioning Clinical Nurse Specialists and Nurse Practitioners as Change Champions to Implement a Pain Protocol in Long-Term Care. *Pain Management Nursing*, *16*(2), 78–88. https://dx.doi.org/10.1016/j.pmn.2014.04.002

Kauth, M. R., Sullivan, G., Blevins, D., Cully, J. A., Landes, R. D., Said, Q., & Teasdale, T. A. (2010). Employing External Facilitation to Implement Cognitive Behavioral Therapy in VA

Clinics: A Pilot Study. *Implementation Science*, *5*(Article 75). https://dx.doi.org/10.1186/1748-5908-5-75

Kelly, J. A., Somlai, A. M., DiFranceisco, W. J., Otto-Salaj, L. L., McAuliffe, T. L., Hackl, K. L., … Rompa, D. (2000). Bridging the Gap Between the Science and Service of HIV Prevention: Transferring Effective Research-Based HIV Prevention Interventions to Community AIDS Service Providers. *American Journal of Public Health*, *90*(7), 1082–1088. https://dx.doi.org/10.2105/ajph.90.7.1082

Kinley, J., Stone, L., Dewey, M., Levy, J., Stewart, R., McCrone, P., … Hockley, J. (2014). The Effect of Using High Facilitation when Implementing the Gold Standards Framework in Care Homes Programme: A Cluster Randomised Controlled Trial. *Palliative Medicine*, *28*(9), 1099–1109. https://dx.doi.org/10.1177/0269216314539785

Kirchner, J. E., Ritchie, M. J., Pitcock, J. A., Parker, L. E., Curran, G. M., & Fortney, J. C. (2014). Outcomes of a Partnered Facilitation Strategy to Implement Primary Care–Mental Health. *Journal of General Internal Medicine*, *29*(Suppl 4), 904–912. https://dx.doi.org/10.1007/s11606-014-3027-2

Kousgaard, M. B., & Thorsen, T. (2012). Positive Experiences with a Specialist as Facilitator in General Practice. *Danish Medical Journal*, *59*(6), A4443. Retrieved from: https://ugeskriftet.dk/files/scientific_article_files/2018-12/a4443.pdf

Kristensen, H. K., & Hounsgaard, L. (2013). Implementation of Coherent, Evidence-Based Pathways in Danish Rehabilitation Practice. *Disability and Rehabilitation*, *35*(23), 2021–2028. https://dx.doi.org/10.3109/09638288.2013.768301

Lavoie-Tremblay, M., Richer, M., Marchionni, C., Cyr, G., Biron, A. D., Aubry, M., … Vézina, M. (2012). Implementation of Evidence-Based Practices in the Context of a Redevelopment Project in a Canadian Healthcare Organization. *Journal of Nursing Scholarship*, *44*(4), 418–427. https://dx.doi.org/10.1111/j.1547-5069.2012.01480.x

Leathers, S. J., Spielfogel, J. E., Blakey, J., Christian, E., & Atkins, M. S. (2015). The Effect of a Change Agent on Use of Evidence-Based Mental Health Practices. *Administration and Policy in Mental Health and Mental Health Services Research*, *43*(5), 768–782. https://dx.doi.org/10.1007/s10488-015-0694-1

Leeman, J., Myers, A., Grant, J. C., Wangen, M., & Queen, T. L. (2017). Implementation Strategies to Promote Community-Engaged Efforts to Counter Tobacco Marketing at the Point of Sale. *Translational Behavioral Medicine*, *7*(3), 405–414. https://dx.doi.org/10.1007/s13142-017-0489-x

Lemelin, J., Hogg, W., & Baskerville, N. (2001). Evidence to Action: A Tailored Multifaceted Approach to Changing Family Physician Practice Patterns and Improving Preventive Care. *Canadian Medical Association Journal*, *164*(6), 757–763. Retrieved from https://www.cmaj.ca/content/164/6/757.short

Lessard, S., Bareil, C., Lalonde, L., Duhamel, F., Hudon, E., Goudreau, J., & Lévesque, L. (2016). External Facilitators and Interprofessional Facilitation Teams: A Qualitative Study of Their Roles in Supporting Practice Change. *Implementation Science*, *11*(Article 97). https://dx.doi.org/10.1186/s13012-016-0458-7

Mackenzie, T., Innes, J., Boyd, M., Keane, B., Boxall, J., & Allan, S. (2011). Evaluating the Role and Value of a National Office to Coordinate Liverpool Care Pathway Implementation in New Zealand. *International Journal of Evidence-Based Healthcare*, *9*, 252–260.

https://dx.doi.org/10.1111/j.1744-1609.2011.00219.x

Mader, E. M., Fox, C. H., Epling, J. W., Noronha, G. J., Swanger, C. M., Wisniewsk, A. M., … Morley, C. P. (2016). A Practice Facilitation and Academic Detailing Intervention Can Improve Cancer Screening Rates in Primary Care Safety Net Clinics. *Journal of the American Board of Family Medicine*, *29*(5). https://dx.doi.org/10.3122/jabfm.2016.05.160109

Mancini, A. D., Moser, L. L., Whitley, R., McHugo, G. J., Bond, G. R., Finnerty, M. T., & Burns, B. J. (2009). Assertive Community Treatment: Facilitators and Barriers to Implementation in Routine Mental Health Settings. *Psychiatric Services*, *60*(2), 189–195. https://dx.doi.org/10.1176/ps.2009.60.2.189

McCullough, M. B., Gillespie, C., Petrakis, B. A., Jones, E. A., Park, A. M., Lukas, C. V., & Rose, A. J. (2017). Forming and Activating an Internal Facilitation Group for Successful Implementation: A Qualitative Study. *Research in Social and Administrative Pharmacy*, *13*(5), 1014–1027. https://dx.doi.org/10.1016/j.sapharm.2017.04.007

Meropol, S. B., Schiltz, N. K., Sattar, A., Stange, K. C., Nevar, A. H., Davey, C., … Cuttler, L. (2014). Practice-Tailored Facilitation to Improve Pediatric Preventive Care Delivery: A Randomized Trial. *Pediatrics*, *133*(6), e1664–e1675. https://dx.doi.org/10.1542/peds.2013-1578

Mitchell, R. E., Stone-Wiggins, B., Stevenson, J. F., & Florin, P. (2004). Cultivating Capacity. *Journal of Prevention & Intervention in the Community*, *27*(2), 67–87. https://dx.doi.org/10.1300/j005v27n02_05

Mold, J. W., Aspy, C. A., & Nagykaldi, Z. (2008). Implementation of Evidence-Based Preventive Services Delivery Processes in Primary Care: An Oklahoma Physicians Resource/Research Network (OKPRN) Study. *The Journal of the American Board of Family Medicine*, *21*(4), 334–344. https://dx.doi.org/10.3122/jabfm.2008.04.080006

Mold, J. W., Fox, C., Wisniewski, A., Lipman, P. D., Krauss, M. R., Harris, D. R., … Gonin, R. (2014). Implementing Asthma Guidelines Using Practice Facilitation and Local Learning Collaboratives: A Randomized Controlled Trial. *The Annals of Family Medicine*, *12*(3), 233–240. https://dx.doi.org/10.1370/afm.1624

Murray, M. E., Khoury, D. Y., Farmer, E. M. Z., & Burns, B. J. (2018). Is More Better? Examining Whether Enhanced Consultation / Coaching Improves Implementation. *American Journal of Orthopsychiatry*, *88*(3), 376–385. https://dx.doi.org/10.1037/ort0000296

Nadeem, E., Gleacher, A., Pimentel, S., Hill, L. C., McHugh, M., & Hoagwood, K. E. (2013). The Role of Consultation Calls for Clinic Supervisors in Supporting Large-Scale Dissemination of Evidence-Based Treatments for Children. *Administration and Policy in Mental Health and Mental Health Services Research*, *40*(6), 530–540. https://dx.doi.org/10.1007/s10488-013-0491-7

Olson, J. R., McCarthy, K. J., Perkins, D. F., & Borden, L. M. (2018). A Formative Evaluation of a Coach-Based Technical Assistance Model for Youth- and Family-Focused Programming. *Evaluation and Program Planning*, *67*, 29–37. https://dx.doi.org/10.1016/j.evalprogplan.2017.11.002

Palinkas, L. A., Saldana, L., Chou, C.-P., & Chamberlain, P. (2017). Use of Research Evidence and Implementation of Evidence-Based Practices in Youth-Serving Systems. *Children and Youth Services Review*, *83*, 242–247. https://dx.doi.org/10.1016/j.childyouth.2017.11.005

Parchman, M. L., Noel, P. H., Culler, S. D., Lanham, H. J., Leykum, L. K., Romero, R. L., & Palmer, R. F. (2013). A Randomized Trial of Practice Facilitation to Improve the Delivery of Chronic Illness Care in Primary Care: Initial and Sustained Effects. *Implementation Science*, *8*(Article 93). https://dx.doi.org/10.1186/1748-5908-8-93

Peterson, D. J., Christiansen, A. L., Guse, C. E., & Layde, P. M. (2015). Community Translation of Fall Prevention Interventions: The Methods and Process of a Randomized Trial. *Journal of Community Psychology*, *43*(8), 1005–1018. https://dx.doi.org/10.1002/jcop.21728

Preast, J. L., & Burns, M. K. (2018). Effects of Consultation on Professional Learning Communities. *Journal of Educational and Psychological Consultation*, *29*(2), 1–31. https://dx.doi.org/10.1080/10474412.2018.1495084

Proctor, E. K., Hooley, C., Morse, A., McCrary, S., Kim, H., & Kohl, P. L. (2019). Intermediary / Purveyor Organizations for Evidence-Based Interventions in the US Child Mental Health: Characteristics and Implementation Strategies. *Implementation Science*, *14*(Article 3). https://dx.doi.org/10.1186/s13012-018-0845-3

Quanbeck, A., Brown, R., Zgierska, A., Jacobson, N., Robinson, J., Johnson, R., Deyo, B., Madden, L., Tuan, W., Alagoz, E. (2018). A Randomized Matched-Pairs Study of Feasibility, Acceptability, and Effectiveness of Systems Consultation: A Novel Implementation Strategy for Adopting Clinical Guidelines for Opioid Prescribing in Primary Care. *Implementation Science*, *13*(Article 21). https://dx.doi.org/10.1186/s13012-018-0713-1

Ritchie, M., Parker, L., Edlund, C., Kirchner, J. (2017). Using Implementation Facilitation to Foster Clinical Practice Quality and Adherence to Evidence in Challenged Settings: A Qualitative Study. *BMC Health Services Research*, *17*(Article 294). https://dx.doi.org/10.1186/s12913-017-2217-0

Rivard, L. M., Russell, D. J., Roxborough, L., Ketelaar, M., Bartlett, D. J., & Rosenbaum, P. (2010). Promoting the Use of Measurement Tools in Practice: A Mixed-Methods Study of the Activities and Experiences of Physical Therapist Knowledge Brokers. *Physical Therap*, *90*(11), 1580–1590. https://dx.doi.org/10.2522/ptj.20090408

Rohrbach, L. A., Gunning, M., Sun, P., & Sussman, S. (2010). The Project Toward No Drug Abuse (TND) Dissemination Trial: Implementation Fidelity and Immediate Outcomes. *Prevention Science*, *11*(1), 77–88. https://dx.doi.org/10.1007/s11121-009-0151-z

Rosella, L., Bornbaum, C., Kornas, K., Lebenbaum, M., Peirson, L., Fransoo, R., Loeppky, C., Gardner, C., Mowat, D. (2018). Evaluating the Process and Outcomes of a Knowledge Translation Approach to Supporting Use of the Diabetes Population Risk Tool (DPoRT) in Public Health Practice. *Canadian Journal of Program Evaluation*, *33*(1), 21-48. https://dx.doi.org/10.3138/cjpe.31160

Rosen, C. S., Nguyen, T., Bernardy, N. C., Hamblen, J. L., Ruzek, J. I., & Friedman, M. J. (2012). Evaluation of a Mentoring Program for PTSD Clinic Managers in the U.S. Department of Veterans Affairs. *Psychiatric Services*, *63*(10), 1047–1050. https://dx.doi.org/10.1176/appi.ps.201100446

Rushovich, B., Bartley, L., Steward, R., Bright, C. (2015). Technical Assistance: A Comparison Between Providers and Recipients. *Human Service Organizations: Management, Leadership & Governance*, *39*(4), 362-379. https://dx.doi.org/10.1080/23303131.2015.1052124

Russell, D., Rivard, L., Walter, S., Rosenbaum, P., Roxborough, L., Cameron, D., Darrah, J., Bartlett, D., Hanna, S., Avery, L. (2010). Using Knowledge Brokers to Facilitate the Uptake of Pediatric

Measurement Tools into Clinical Practice: A Before-After Intervention Study. *Implementation Science*, *5*(Article 92). https://dx.doi.org/10.1186/1748-5908-5-92

Ryba, M. M., Brothers, B. M., & Andersen, B. L. (2017). Implementation of an Evidence-Based Biobehavioral Treatment for Cancer Patients. *Translational Behavioral Medicine*, *7*, 648–656. https://dx.doi.org/10.1007/s13142-016-0459-8

Saldana, L., & Chamberlain, P. (2012). Supporting Implementation: The Role of Community Development Teams to Build Infrastructure. *American Journal of Community Psychology*, *50*(3–4), 334–346. https://dx.doi.org/10.1007/s10464-012-9503-0

Sanetti, L. M. H., Fallon, L. M., & Collier-Meek, M. A. (2013). Increasing Teacher Treatment Integrity Through Performance Feedback Provided by School Personnel. *Psychology in the Schools*, *50*(2), 134–150. https://dx.doi.org/10.1002/pits.21664

Sanetti, L. M. H., Williamson, K. M., Long, A. C. J., & Kratochwill, T. R. (2018). Increasing In-Service Teacher Implementation of Classroom Management Practices Through Consultation, Implementation Planning, and Participant Modeling. *Journal of Positive Behavior Interventions*, *20*(1), 43–59. https://dx.doi.org/10.1177/1098300717722357

Seers, K., Rycroft-Malone, J., Cox, K., Crichton, N., Edwards, R., Eldh, A., Estabrooks, C., Harvey, G., Hawkes, C., Jones, C., Kitson, A., McCormack, B., McMullan, C., Mockford, C., Niessen, T., Slater, P., Titchen, A., Zijpp, T., Wallin, L. (2018). Facilitating Implementation of Research Evidence (FIRE): An International Cluster Randomised Controlled Trial to Evaluate two Models of Facilitation Informed by the Promoting Action on Research Implementation in Health Services (PARIHS) Framework. *Implementation Science, 13*(Article 137). https://dx.doi.org/10.1186/s13012-018-0831-9

Shernoff, E. S., Lakind, D., Frazier, S. L., & Jakobsons, L. (2015). Coaching Early Career Teachers in Urban Elementary Schools: A Mixed-Method Study. *School Mental Health*, *7*(1), 6–20. https://dx.doi.org/10.1007/s12310-014-9136-6

Stirman, S. W., Pontoski, K., Creed, T., Xhezo, R., Evans, A. C., Beck, A. T., & Crits-Christoph, P. (2017). A Non-Randomized Comparison of Strategies for Consultation in a Community-Academic Training Program to Implement an Evidence-Based Psychotherapy. *Administration and Policy in Mental Health and Mental Health Services Research*, *44*(1), 55–66. https://dx.doi.org/10.1007/s10488-015-0700-7

Tierney, S., Kislov, R., Deaton, C. (2014). A Qualitative Study of a Primary-Care Based Intervention to Improve the Management of Patients with Heart Failure: The Dynamic Relationship Between Facilitation and Context. *BMC Family Practice*, *15*(Article 153). https://dx.doi.org/10.1186/1471-2296-15-153

Van der Zijpp, T. J., Niessen, T., Eldh, A. C., Hawkes, C., McMullan, C., Mockford, C., … Seers, K. (2016). A Bridge Over Turbulent Waters: Illustrating the Interaction Between Managerial Leaders and Facilitators When Implementing Research Evidence. *Worldviews on Evidence-Based Nursing*, *13*(1), 25–31. https://dx.doi.org/10.1111/wvn.12138

Ward, M. M., Baloh, J., Zhu, X., & Stewart, G. L. (2017). Promoting Action on Research Implementation in Health Services Framework Applied to TeamSTEPPS Implementation in Small Rural Hospitals. *Health Care Management Review*, *42*(1), 2–13. https://dx.doi.org/10.1097/hmr.0000000000000086

Waterman, H., Boaden, R., Burey, L., Howells, B., Harvey, G., Humphreys, J., Rothwell, K., Spence, M. (2015). Facilitating Large-Scale Implementation of Evidence-Based Health Care: Insider

Accounts from a Co-Operative Inquiry. *BMC Health Services Research*, *15*(Article 60). https://dx.doi.org/10.1186/s12913-015-0722-6

Williams, L., Burton, C., & Rycroft-Malone, J. (2013). What Works: A Realist Evaluation Case Study of Intermediaries in Infection Control Practice. *Journal of Advanced Nursing*, *69*(4), 915–926. https://dx.doi.org/doi: 10.1111/j.1365-2648.2012. 06084.x

Williams, L., Daggett, V., Slaven, J. E., Yu, Z., Sager, D., Myers, J., … Damush, T. M. (2016). A Cluster-Randomised Quality Improvement Study to Improve Two Inpatient Stroke Quality Indicators. *BMJ Quality & Safety*, *25*, 257–264. https://dx.doi.org/http://dx.doi.org/10.1136/ bmjqs-2015-004862

Williams, N. J., Glisson, C., Hemmelgarn, A., & Green, P. (2017). Mechanisms of Change in the ARC Organizational Strategy: Increasing Mental Health Clinicians' EBP Adoption Through Improved Organizational Culture and Capacity. *Administration and Policy in Mental Health and Mental Health Services Research*, *44*, 269–283. https://dx.doi.org/10.1007/s10488-016-0742-5

Worton, S. K., Hasford, J., Macnaughton, E., Nelson, G., MacLeod, T., Tsemberis, S., … Richter, T. (2018). Understanding Systems Change in Early Implementation of Housing First in Canadian Communities: An Examination of Facilitators / Barriers, Training / Technical Assistance, and Points of Leverage. *American Journal of Community Psychology*, *61*(1–2), 118–130. https://dx.doi.org/10.1002/ajcp.12219

Yano, E. M., Rubenstein, L. V., Farmer, M. M., Chernof, B. A., Mittman, B. S., Lanto, A. B., … Sherman, S. E. (2008). Targeting Primary Care Referrals to Smoking Cessation Clinics Does Not Improve Quit Rates: Implementing Evidence-Based Interventions into Practice. *Health Services Research*, *43*, 1637–1661. https://dx.doi.org/10.1111/j.1475-6773.2008.00865.x

Yazejian, N., Metz, A., Morgan, J., Louison, L., Bartley, L., Fleming, W., Haidar, L., Schroeder, J. (2019). Co-Creative Technical Assistance: Essential Functions and Interim Outcomes. *Evidence & Policy: A Journal of Research, Debate and Practice*, 15(3), 339-352. https://dx.doi.org/10.1332/174426419x15468578679853
