# Supplementary material for: Knowledge and attitudes of Implementation Support Practitioners—Findings from a systematic integrative review
Source: PLoS One. 2022 May 11;17(5):e0267533. doi: 10.1371/journal.pone.0267533 (PMC9094539; doi:10.1371/journal.pone.0267533)
Supplement: S3 Appendix — (PDF) [file pone.0267533.s003.pdf]

S3 Appendix: Overview of the included studies*

Included Publications – Aims, Design, Geography, Sectors and Settings

REF	Publication	ISP Role	Aim	Design	Country	Sector	Setting	Knowledge	Attitudes
[94]	Aasekjær 2016	Non-specified	To gain knowledge about factors contributing to the implementation of EBP among health professionals who attended a post-professional EBP program.	Qual	Norway	Health	University hospitals	√	√
[47]	Acolet 2011	Other	To assess whether an active strategy for the dissemination of neonatal research findings, recommendations and guidelines was more likely to change policy and practice than the passive dissemination of a report, a slide package, and a position statement.	Quan	U.K.	Health	Maternal & child health	√	
[81]	Akin 2016	Coach	To articulate and understand the core functions of coaching from the perspective of coaches and child welfare workers.	Qual	U.S.	SW	Child welfare agency		√
[48]	Anaby 2015	Knowledge Broker	(A) To identify site-specific needs and issues of clinicians working with children youth with physical disabilities to facilitate child participation AND (B) To develop and evaluate an intervention plan to facilitate knowledge uptake by clinicians to foster change in practice behavior, specifically in the topic of child participation.	Qual	Canada	Health	Pediatric rehabilitation providers	√	
[95]	Barac 2018	Consultant	To examine consultation in the context of implementing Motivational Interviewing (MI) in four community child and youth health organizations in Canada with a goal to describe the consultation process and explore clinicians' and supervisors' perspectives on it.	Qual	Canada	Health	Community mental health	√	√
[96]	Becker 2013	Coach	To examine how coaches tailored their coaching practices to teacher implementation quality of the Good Behavior Game (GBG); and to explore the association between coaching and the implementation of the GBG by elementary school teachers.	Quan	U.S.	EDU	Elementary school classrooms (K-5)	√	√
[49]	Beidas 2012a	Consultant	To evaluate the efficacy of three different brief CBT training modalities, and to evaluate the impact of including ongoing consultation after this training on therapist CBT knowledge, skill and adherence and training satisfaction.	Quan	U.S.	Health	Mixed -Schools, outpatient clinics, hospital, community mental agencies	√	
[82]	Beidas 2013	Consultant	To identify what therapists who participated in a training and consultation study two years prior perceived to be the active ingredients of consultation.	Qual	U.S.	Health	Community mental health		√
[97]	Bidassie 2015	Facilitator	To clarify and explain the facilitation techniques, i.e.,	Qual	U.S.	Health	VA medical centers	√	√

REF	Publication	ISP Role	Aim	Design	Country	Sector	Setting	Knowledge	Attitudes
			activities, processes, challenges used and experienced with frontline stroke clinicians and teams to promote quality improvement; and to identify the core components of facilitation.						
[50]	Bradshaw 2012	Coach	To compare student outcomes achieved by PBIS schools that receive (a) traditional training and support in tier 1 implementation from the state and district, and (b) tier 2 training and coaching from an external tier 2 coach (i.e., PBISplus = intervention condition) with those from PBIS schools that receive traditional training and support in tier 1 implementation from the state and district only (i.e., SWPBIS = comparison/ control condition).	Quan	U.S.	EDU	Elementary schools	√	
[51]	Brown 2018a	Knowledge Broker	To answer the following research questions: (a) Which factors influenced schools' participation in COMPASS knowledge brokering?, and (b) Did participating in knowledge brokering influence changes in school health policies and practices and/or student health behaviors?	Quan	Canada	Health	Secondary schools	√	
[83]	Brown 2018c	Knowledge Broker	To explore researchers' and knowledge users' experience with COMPASS knowledge exchange activities with a focus on perceived outcomes and suggestions for change	Qual	Canada	Health	Secondary Schools		√
[52]	Byrnes 2018	Facilitator	To assess implementation outcomes and changes in clinical process outcomes achieved through the use of a theory-informed, facilitated implementation intervention aimed at improving adherence to evidence-based post-operative diet guidelines used with older surgical patients.	MM	Australia	Health	General surgical wards at a metropolitan tertiary teaching hospital	√	
[98]	Cameron 2011	Knowledge Broker	To explore administrators' perspectives on the successes and challenges of having a KB in their agency to facilitate more evidence-based practice, and their perception of the generalizability of the KB process to meet other EBP needs.	Qual	Canada	Health	Children's rehabilitation organizations	√	√
[53]	Caron 2019	Consultant	To examine the effects of adding fidelity consultation to coaching as usual on the implementation of Attachment and Biobehavioral Catch-Up (ABC) amongst community-based clinicians in Hawaii.	Quan	U.S.	SW	Community provider agencies	√	
[99]	Chaffin 2016	Imp Team	To examine the SafeCare fidelity trajectories for members of the interagency collaborative ("seed") team and the first cohort of SafeCare providers trained and coached by this team.	Quan	U.S.	SW	Community provider agencies	√	√
[84]	Chew 2013	Intermediary	To explore full-time intermediaries from the role-holders' standpoint; examine the impact of enacting such roles on	Qual	U.K.	Health	One CLAHRC (= multiple NHS provider		√

REF	Publication	ISP Role	Aim	Design	Country	Sector	Setting	Knowledge	Attitudes
			the individual itself; and to explore the potential impact of these individual consequences for the longer-term viability of intermediary roles as a knowledge-translation solution.				organizations)		
[54]	Chinman 2017	TA Provider	To evaluate how much a Getting to Outcomes (GTO) guided implementation strategy (training + manuals + implementation support) facilitated the adoption and implementation of the MISSION-Vet intervention targeting (formerly) homeless veterans – when compared with implementation as usual (training + manuals only).	MM	U.S.	Health	VA medical centers	√	
[55]	Chinman 2018	TA Provider	To assess the effectiveness of the GTO Implementation Support Intervention by comparing the outcomes from 15 Boys and Girls Club sites implementing CHOICE (control group), a five-session evidence-based alcohol and drug prevention program, with 14 similar sites implementing CHOICE supported by GTO (intervention group).	Quan	U.S.	Health	Community provider agencies	√	
[56]	Dobbins 2018	Knowledge Broker	To develop a tailored knowledge translation strategy and to test it through case studies to observe impacts on evidence-informed decision-making (EIDM) knowledge, skills, and behaviour, and identify contextual factors that were barriers or facilitators.	MM	Canada	Health	Three public health departments in Ontario	√	
[100]	Dogherty 2012	Facilitator	To understand the nature of facilitation in supporting the use of a systematic methodology to adapt existing clinical practice guidelines for Canadian use.	MM	Canada	Health	N/A	√	√
[101]	Dogherty 2013	Facilitator	To describe nurses' tacit knowledge of and perspective on facilitation within contexts of evidence-based practice settings.	Qual	Canada	Health	Different nursing work contexts	√	√
[57]	Duffy 2012	TA Provider	To answer three research questions: (1) Does the delivery of intense and proactive training and technical assistance (T/TA) increase community service agencies' capacity to use an adapted version of the Getting-to-Outcomes (GTO) framework with their teen pregnancy prevention programs? (2) What were facilitating factors for an increase in capacity? (3) What were barriers to an increase in capacity?	Qual	U.S.	Health	Community agencies & schools	√	
[102]	Dusenbury 2010	Coach	To explore and describe the topics coaches covered with teachers during coaching sessions. To investigate how teachers responded to coaching. To examine whether coaching on various topics produced improvements in student outcomes (mediators and behaviors). To formulate hypotheses for how teachers develop the skill and	MM	U.S.	EDU	Middle schools	√	√

REF	Publication	ISP Role	Aim	Design	Country	Sector	Setting	Knowledge	Attitudes
			understanding to become effective implementers of prevention programs.						
[103]	Eiraldi 2018	Consultant	To test which of two consultation strategy (Consultation versus Consultation+) used with school counselors resulted in (a) higher treatment content and process fidelity of an intervention aimed at externalizing behaviours in school children (CPP); and (b) better child outcomes (changes in diagnostic status and impairment).	MM	U.S.	EDU	Under-resourced, urban schools	√	√
[104]	Elnitsky 2015	Facilitator	To understand internal facilitation activities in implementing a national safe patient handling program from the perspective of facility coordinators who implemented the program.	Qual	U.S.	Health	VA medical centers	√	√
[58]	Feinberg 2008	TA Provider	To understand the impact of dosage of on-site and off-site technical assistance on the functioning of Communities That Care prevention boards.	Quan	U.S.	CJ	Communities (CTC sites)	√	
[59]	Fortney 2018	Imp Team	To understand the impact of an external facilitation method – evidence-based quality improvement (EBQI) – on the implementation of evidence-based practices for bi-polar depression and treatment-resistant depression in health centres.	Quan	U.S.	Health	Federally qualified health centres	√	
[60]	Funderburk 2015	Consultant	To answer the following research question: Does live video consultation improve downstream client outcomes relative to standard phone consultation?	Quan	U.S.	Health	Community-based health providers	√	
[61]	Garbacz 2016	Consultant	To investigate how Conjoint Behavioural Consultation (CBC) can be used to provide EBP mental health services in schools.	Quan	U.S.	Health	K8-schools in low-income areas	√	
[105]	Gerrish 2011	Knowledge Broker	To identify how APNs (advanced practice nurse) promote EBP amongst CNs (clinical nurse).	Qual	U.K.	Health	Primary care practices & hospitals	√	√
[106]	Gerrish 2012	Knowledge Broker	To identify factors that influence APNs (advanced practice nurse) ability to promote EBP among FLNs (frontline nurse).	Qual	U.K.	Health	Primary care practices & hospitals	√	√
[107]	Graaf 2017	Knowledge Broker	To increase the understanding of the role of the Link Officer (LO) and Link PARTner (LP) in supporting evidence-informed practice within their Health Service Organizations by answering the following research questions: (a) What are the professional characteristics of individuals serving in LP/LO roles? (b) What do LPs/LOs understand their role to entail and what major activities are involved in fulfilling those responsibilities? (c) How well are the professional efforts of LPs/LOs supported by their own organizations and	MM	Mix	SW	Child welfare and family service provider agencies	√	√

REF	Publication	ISP Role	Aim	Design	Country	Sector	Setting	Knowledge	Attitudes
			their intermediary organizations?						
[108]	Gunderson 2018	Coach	To examine (a) how stakeholder perspectives on and experience with coaching changed across implementation phases, and (b) the factors influencing coaching when used to support the implementation of SafeCare.	Qual	U.S.	SW	Community provider agencies	√	√
[62]	Gustafson 2013	Mix	To test the effectiveness of four different quality improvement interventions to reduce patients' waiting time, retention and to increase the number of patients served annually across addiction clinics in the U.S.	Quan	U.S.	Health	Outpatient addiction treatment clinics	√	
[63]	Hagermoser Sanetti 2013	Consultant	1) to test whether performance feedback effectively increase teachers' level of treatment integrity when implementing a class wide, team-based contingency management intervention, when delivered by an internal consultant and 2) to assess at what level of treatment integrity school-based personnel are able to implement performance feedback procedures.	Quan	U.S.	EDU	Suburban middle school	√	
[64]	Hagermoser Sanetti 2018	Consultant	To evaluate the effectiveness of implementation planning (logistical planning and identification of implementation barriers), and participant modelling (didactive and in vivo intervention training) on teachers' implementation of an evidence-based classroom behavioral management intervention.	Quan	U.S.	EDU	Elementary schools	√	
[65]	Hendricks Brown 2014	Facilitator	To determine whether participation in the Community Development Team (CDT) model improves the rates of program adoption, implementation, and fidelity of Multidimensional Treatment Foster Care (MTFC) in 51 counties (56 sites) in California and Ohio when compared with an individualized implementation condition (IND).	Quan	U.S.	SW	Child public service systems	√	
[66]	Holtrop 2008	Consultant	To describe and examine the process of nurse consultation and how it might be used to implement changes in primary care practices.	Qual	U.S.	Health	Primary care practices	√	
[67]	Hurlburt 2014	Imp Team	To understand how the interagency collaborative team (ICT) implementation process model generated key structural supports for the implementation of SafeCare, and to identify process issues that require further consideration.	Qual	U.S.	SW	Community organisations	√	
[85]	Hurtubise 2016	Knowledge Broker	To describe the roles identified by knowledge brokers (KBs) working within virtual communities of practice (vCoP). Specifically, to better understand the roles played by KBs within the vCoP, the work activities performed to enact	Qual	Canada	Health	Pediatric rehabilitation providers		√

REF	Publication	ISP Role	Aim	Design	Country	Sector	Setting	Knowledge	Attitudes
			those roles, their role evolution, and the facilitation strategies they used over a 5-month period.						
[109]	Jacobson 2018	Consultant	To (1) describe how systems consultation – a blended implementation strategy used to promote guideline-adherent opioid describing practices - worked during a pilot test and (2) identify the modifications necessary to adapt this implementation strategy to primary care.	Qual	U.S.	Health	Community-based primary care clinics	√	√
[86]	Kaasalainen 2015	Imp Team	To answer the following research questions: (1) How do Nurse Practitioners and Clinical Nurse Specialist facilitate effective change in practice to enable the implementation of a pain protocol in long term care? (2) What barriers and facilitators are encountered by the Clinical Nurse Specialist and Nurse Practitioners in changing team practice to implement this protocol?	Qual	Canada	Health	Long term care facilities		√
[87]	Kauth 2010	Facilitator	To examine the effect of facilitation on the implementation of cognitive behavioral therapy (CBT), an evidence-based therapy, in Department of Veteran Affairs clinics.	MM	U.S.	Health	Primary care practices		√
[68]	Kelly 2000	Consultant	Compare the effectiveness of 3 dissemination strategies for transferring HIV prevention models from the research arena to community providers of HIV prevention services.	MM	U.S.	Health	AIDS service organizations (ASOs)	√	
[69]	Kirchner 2014	Facilitator	To test the hypothesis that, compared to national implementation support alone, national support plus a partnered implementation facilitation strategy would improve the implementation of Primary Care-Mental Health Integration across multiple Veterans Affairs in the U.S..	MM	U.S.	Health	VA primary care clinics	√	
[110]	Kousgaard 2012	Strategy use	To explore the experience and assessment of GPs and nurses participating in a project in which a medical specialist (endocrinologist) acted as a facilitator for quality improvement.	Qual	Denmark	Health	Primary care practices	√	√
[88]	Kristensen 2013	Facilitator	To gain a deeper understanding of how to facilitate the implementation of coherent and evidence-based guidelines in Danish rehabilitation contexts.	Qual	Denmark	Health	Community provider agencies & hospitals		√
[98]	Lavoie-Tremblay 2012	Knowledge Broker	To understand how a Project management office (PMO) facilitates successful implementation of evidence-based practice in care delivery.	Qual	Canada	Health	University affiliated multi-site health care center		√
[111]	Leathers 2016	Consultant	To investigate the effect of post-training change agent interactions on the use of an evidence-based intervention relative to training as usual with no additional post-training support.	Quan	U.S.	SW	Child welfare agency	√	√

REF	Publication	ISP Role	Aim	Design	Country	Sector	Setting	Knowledge	Attitudes
[70]	Leeman 2017	TA Provider	To explore how the use of 'Counter Tools' – a provider of tools, training, and TA to enhance the implementation of evidence-based policies aimed at countering tobacco marketing at points of sale – changed community coordinators' self-efficacy, partnerships' performance of core policy change processes, and communities' progress toward EBPI enactment after 1 year. And: To identify contextual barriers to progress towards local EBPI enactment.	MM	U.S.	Health	Community agencies & local health departments	√	
[90]	Lessard 2016	Facilitator	To 1) identify and analyze the facilitation roles undertaken by external facilitators (EFs) and interprofessional facilitation teams (IFTs) during the implementation of TRANSIT; and (2) examine the dynamics of facilitation between EFs, IFTs, family medicine groups, and other change actors.	Qual	Canada	Health	Primary care practices		√
[91]	Mackenzie 2011	Strategy use	To evaluate the role and value of the New Zealand National LCP Office (Liverpool Care Pathway for the Dying Patient) (NZ LCP) from the perspective of key stakeholders.	MM	New Zealand	Health	Community and residential care facilities, hospices, hospitals		√
[71]	Mader 2016	Facilitator	To evaluate the efficacy and feasibility of combining practice facilitation and academic detailing to help primary care practices increase patient breast cancer, cervical cancer, and colorectal cancer screening.	MM	U.S.	Health	Primary care practices	√	
[112]	Mancini 2009	Consultant	To assess the treatment fidelity of 13 teams that had begun to implement Assertive Community Treatment (ACT) in two states in the U.S, and to identify barriers and facilitators to the high-fidelity implementation of ACT.	MM	U.S.	Health	Community agencies & hospitals	√	√
[113]	McCullough 2017	Facilitator	To examine the roles and actions of an internal facilitation team in a regional anti-coagulation improvement initiative involving eight clinics.	Qual	U.S.	Health	VA medical centers' anticoagulation clinics	√	√
[114]	Mitchell 2004	TA Provider	To answer the following research questions: (1) What are the conditions under which prevention community coalitions (PCC) will utilize available technical assistance? (2) What are the effects of this TA on PCC intermediate outcomes (e.g., coalition functioning, programming etc.)?	Quan	U.S.	Mix	Prevention community coalitions	√	√
[72]	Murray 2018	Consultant	To examine whether the initial model of Together facing the Challenge (TFTC) could be improved to increase sustainability and improve implementation.	Quan	U.S.	SW	Private non-profit agencies	√	
[73]	Nadeem 2013	Consultant	To explore the content and process of consultation provided to clinic supervisors supporting therapists who implement	Qual	U.S.	SW	Outpatient community clinics	√	

REF	Publication	ISP Role	Aim	Design	Country	Sector	Setting	Knowledge	Attitudes
			evidence-based programs targeting children with disruptive behavior disorders and are part of a statewide training program through which they – next to their clinical supervision – also received external consultation.				and other settings		
[92]	Olson 2018	Mix	To present results from a preliminary formative evaluation examining factors related to the acceptance of a coaching model used in the Children, Youth and Families at Risk Initiative (CYFAR), aimed at promoting the development and delivery of evidence-informed educational programs designed to meet the basic needs of youth and families considered to be at risk for various negative outcomes.	Quan	U.S.	SW	N/A		√
[74]	Parchman 2013	Facilitator	To assess the effectiveness of practice facilitation to improve the organization and delivery of diabetes care in small, autonomous primary care practices.	Quan	U.S.	Health	Primary care practices (with between 1-3 clinicians)	√	
[75]	Peterson 2015	Mix	To assess whether a prevention support system (i.e., “enhanced support system”) that builds capacity through the provision of technical assistance, best practice guides, and direct consultation can successfully bridge the gap between fall prevention research and the implementation of an evidence-driven, community-based fall prevention program.	Quan	U.S.	Health	Mixed - Health department, local public health department, county public health department, aging units, community coalition	√	
[115]	Quanbeck 2018	Consultant	To pilot test the blended implementation strategy ‘systems consultation’ (SC) in a small set of primary care clinics to see if the strategy demonstrated feasibility, acceptability, and preliminary effectiveness in improving clinician adherence to opioid-prescribing guidelines and reducing morphine-equivalent daily dose (MEDD) for patients on long-term opioid therapy.	MM	U.S.	Health	Community-based primary clinics	√	√
[76]	Ritchie 2017	Facilitator	To assess whether implementation facilitation (IF) could 1) help clinics with challenging contexts to implement PC-MHI programs, and 2) foster development of PC-MHI programs that are of high quality, adhere to evidence, are sustainable and are likely to 3) lead to improvement of clinical practices and outcomes as assessed by experts.	Qual	U.S.	Health	VA primary care clinics integrated into the community or into VA medical centres	√	
[116]	Rivard 2010	Knowledge Broker	To describe the brokering activities of pediatric physical therapist knowledge brokers (KBs) and to report KBs’ perceptions of the utility of their role and their experience with the brokering process.	MM	Canada	Health	Pediatric rehabilitation providers	√	√

REF	Publication	ISP Role	Aim	Design	Country	Sector	Setting	Knowledge	Attitudes
[117]	Rosella 2018	Knowledge Broker	To evaluate the use of the Population Health Planning Knowledge to Action (PHP KtA) Model in promoting the implementation of the Diabetes Population Risk Tool (DPoRT) a health planning tool targeting diabetes services; and to identify strategies helping to use DPoRT, and to remove barriers to using DPoRT.	MM	Canada	Health	Public health provider organizations	√	√
[93]	Rosen 2012	Other	To elicit feedback from participants in a mentoring program intended to help posttraumatic stress disorder (PTSD) clinical managers address organizational challenges in providing evidence-based services to veterans and improving their care.	Quan	U.S.	Health	VA regional health networks		√
[118]	Rushovich 2015	TA Provider	To understand how the technical assistance (TA) provided by a child welfare implementation center was perceived by TA recipients and providers and thereby answer the following research question: What is included in useful TA (i.e., functions, types, strategies, expertise, and frequency) to build state child welfare organizational capacity to support implementation?	Qual	U.S.	SW	Child welfare agency	√	√
[77]	Russell 2010	Knowledge Broker	To evaluate the impact of a multifaceted knowledge translation intervention, using physiotherapists (PTs) as knowledge brokers (KBs) to facilitate the use of four evidence-based measurement tools designed to evaluate and understand motor function in children with cerebral palsy (CP) in clinical practice.	MM	Canada	Health	Children's rehabilitation organizations	√	
[78]	Saldana 2012	Facilitator	To determine whether participation in the Community Development Team (CDT) model improves the rates of program adoption, implementation, and fidelity of Multidimensional Treatment Foster Care (MTFC) in 51 counties (56 sites) in California and Ohio when compared with an individualized implementation condition.	Quan	U.S.	SW	N/A	√	
[119]	Shernoff 2014	Coach	To examine whether a coaching intervention aimed at supporting early career elementary school teachers in classroom management and the engagement of their learners (predictors of teacher attrition) was Implemented as intended; To identify barriers and facilitators to coaching in this setting; To explore whether additional supervision – provided to coaches – was implemented as intended and understand how it was perceived by coaches.	MM	U.S.	EDU	Elementary schools	√	√
[79]	Stirman 2017	Consultant	To compare the effectiveness of two different consultation	Quan	U.S.	Health	Agencies providing	√	

REF	Publication	ISP Role	Aim	Design	Country	Sector	Setting	Knowledge	Attitudes
			strategies - Individual observation and feedback AND Group consultation with feedback – in increasing individual therapists' cognitive therapy (CT) competence, and in maintaining this competence.				general or specialist outpatient services (e.g. substance abuse, mental illness etc.)		
[120]	Tierney 2014	Other	To describe and explore the process of facilitating evidence-based practice in the Greater Manchester Heart Failure Investigation Tool program (GM-HFIT) within the context of primary care and to understand how the process of facilitation is affected by contextual factors.	MM	U.K.	Health	Primary care practices	√	√
[121]	v.d. Zijpp 2016	Facilitator	To describe the interaction between managerial leaders and Internal Facilitators (Ifs) and how this enabled or hindered the facilitation of implementing urinary incontinence guideline recommendations in local service settings providing long-term care to older people.	Qual	Mix	Health	Long-term nursing care provider agencies	√	√
[122]	Waterman 2015	Other	To explore (a) how knowledge transfer associates (KTAs) facilitate the implementation of evidence-based health care in the NHS to improve patient/client care AND (b) how the Greater Manchester "Collaborative for Leadership in Applied Health Research and Care" (GM CLAHRC) managers, clinical leads, and academic leads perceive KTAs facilitate the implementation of evidence-based health care.	Qual	U.K.	Health	Health care commissioning and provider agencies	√	√
[123]	Williams 2013	Intermediar y	To explore and explain how an intermediary program in infection control practice worked in one hospital in the U.K., i.e., generate theoretical explanations about how single intermediaries (i.e., individuals = hospital staff) involved in an intermediary program of a British hospital used specific change processes: What were the mechanisms of change, and for whom were they important? To highlight how these change processes could be transferred to other locations to improve the promotion of evidence-informed infection control practice.	MM	U.K.	Health	Hospitals	√	√
[80]	Williams 2016	Other	To examine the cross-level contextual, motivational, and barrier-related mechanisms that explain the effect of ARC (Availability, Responsiveness, and Continuity implementation support strategy) on clinicians' EBP adoption and use.	Quan	U.S.	Health	Non-profit specialty mental health agencies	√	
[124]	Worton 2018	TA Provider	To understand the early implementation of Housing First (HF), a complex community intervention (CCI) and answer the following research questions: 1. What factors facilitate	MM	Canada	SW	Community provider agencies	√	√

REF	Publication	ISP Role	Aim	Design	Country	Sector	Setting	Knowledge	Attitudes
			or impede planning and early implementation of HF in the exploration and installation stages? 2. How does training and technical assistance (TTA) contribute to HF planning and early implementation? 3. What "levers" for systems change created opportunities for HF planning and early implementation?						
[125]	Yazejian 2019	TA Provider	To examine whether stakeholders from three regions who had utilized technical assistance from a national TA center perceived the TA as co-creative, and whether interim outcomes for a project to increase the use of implementation science concepts were achieved.	MM	U.S.	EDU	Head Start regions	√	√

*Adapted from Albers, B., Bührmann, L., Driessen, P., Bartley, L., & Varsi, C. (2021). Electronic results Addendum (ERA 2): The mechanisms and determinants of implementation support – findings from a systematic integrative review. https://osf.io/7yhm6.
