# Supplementary material for: Knowledge and attitudes of Implementation Support Practitioners—Findings from a systematic integrative review
Source: PLoS One. 2022 May 11;17(5):e0267533. doi: 10.1371/journal.pone.0267533 (PMC9094539; doi:10.1371/journal.pone.0267533)
Supplement: S4 Appendix — The publication IDs indicate the corresponding articles in which the descriptors were identified. (PDF) [file pone.0267533.s004.pdf]

S4 Appendix: Overview of descriptors identified in the included studies categorized in the five knowledge themes (*clinical practice knowledge, implementation / improvement practice knowledge, knowledge about the local context, knowledge about supporting change processes, knowledge about facilitating evidence-based practice in general*). The publication IDs indicate the corresponding articles in which the descriptors were identified.

**Clinical practice**<br><br>*ISPs have knowledge about…*	**Publication references**	**Number of publications**
the intervention and its target population	[48] [49] [54] [55] [57] [59] [60] [61] [64] [65] [66] [67] [68] [70] [72] [73] [75] [79] [95] [96] [97] [99] [102] [103] [104] [106] [108] [109] [110] [111] [117] [119] [120] [122] [123] [124]	n = 36
the research in the clinical area	[47] [48] [50] [51] [57] [66] [68] [69] [70] [71] [74] [95] [98] [103] [104] [105] [106] [109] [121] [122]	n = 20
the field (being clinically experienced and not a novice)	[66] [78] [99] [106] [109] [110] [122]	n = 7
how to assess the needs of a target population and support intervention design	[50] [57] [101]	n = 3
how to administer clinical tools	[116] [117]	n = 2
the principles of sector administration	[118]	n = 1

**Implementation / improvement practice**<br><br>*ISPs have knowledge about…*	**Publication IDs**	**Number of publications**
specific improvement / implementation methods / approaches	[54] [57] [62] [68] [69] [80] [97] [109] [113] [115] [116] [117] [118] [123]	n = 14
the principles of high-quality implementation	[50] [59] [101] [118]	n = 4
the principles of high-quality	[66] [75] [104] [106]	n = 4

facilitation		
one’s own role as an implementation support practitioner	[77] [107] [116] [120]	n = 4
how to guide implementation through appropriate educational materials	[49] [96]	n = 2
an intervention's implementation model / standards and how to maintain these	[95] [96]	n = 2
how to collect and utilize data for intervention design and implementation	[50] [101]	n = 2
how to assess fidelity	[53] [63]	n = 2
how to model and simulate change	[103] [106]	n = 2
implementation strategies	[56] [66]	n = 2
how to select and apply implementation strategies	[98] [104]	n = 2
how to support program adaptation	[57] [59]	n = 2
particular coaching models	[96]	n = 1
coaching models and techniques	[119]	n = 1
how to develop responses to changes in the implementation process	[95]	n = 1
how to assess implementation barriers and plan their removal	[95]	n = 1
how to teach ways to assess fidelity and how to enhance reliable fidelity measurement	[53]	n = 1
how to read and interpret	[53]	n = 1

fidelity data		
how to provide feedback	[103]	n = 1
principles of high-quality coaching	[103]	n = 1
specific consultation interventions	[66]	n = 1
how to make training active	[111]	n = 1
how to provide performance feedback	[63]	n = 1
principles of high-quality consultation	[79]	n = 1
how to assess workflows	[71]	n = 1
one’s own implementation approach	[48]	n = 1
how to evaluate	[117]	n = 1
how to increase demand	[75]	n = 1
how to support program sustainability	[57]	n = 1
typical implementation barriers related to EBP implementation	[57]	n = 1
tools that can support implementation processes (planning templates, measures)	[57]	n = 1
how to collect data	[70]	n = 1
the principles of high-quality evaluation	[114]	n = 1
implementation science	[118]	n = 1

**The local context**<br><br>*ISPs have knowledge about…*	**Publication IDs**	**Number of publications**
organizational structures and	[57] [78] [95] [107] [108] [109] [115] [121]	n = 9

sensitivities	[125]	
local conditions	[58] [66] [69] [71] [119] [122]	n = 6
the local service system	[120] [122] [125]	n = 3
systems used	[71] [122]	n = 2
local practice issues	[107] [116]	n = 2
local policy priorities	[107] [108]	n = 2
how an implementation unfolds locally	[95] [96]	n = 2
local funding structures	[124]	n = 1
local performance	[122]	n = 1
local policies and practices	[47]	n = 1
organizational needs	[107]	n = 1
local constraints	[106]	n = 1
the outer setting	[121]	n = 1
hierarchies, influencers	[52]	n = 1
local key players	[66]	n = 1
how to integrate implementation activities into existing organizational structures	[53]	n = 1
the range of organizations in which an intervention is implemented	[49]	n = 1
parallel implementation initiatives	[95]	n = 1

**Supporting change processes**<br><br>*ISPs have knowledge about…*	**Publication IDs**	**Number of publications**
how to train	[75] [114] [116] [118] [123]	n = 5
how to effectively communicate	[66] [98] [100]	n = 3

how to lead / facilitate groups including participation in group processes, decision-making and conflict resolution	[72] [100] [124]	n = 3
how to solve logistical issues	[103] [109]	n = 2
principles of organizational change	[109] [118]	n = 2
how to enhance skills required for change	[96]	n = 1
ongoing / planned organizational change processes	[95]	n = 1
how to plan and prepare	[60]	n = 1
how to organize (e.g. meetings, collaborative processes)	[100]	n = 1
how to manage time	[98]	n = 1
small group learning approaches	[56]	n = 1
how to lead processes	[106]	n = 1
the principles of organizational / individual behavior change and how to maintain such change	[47]	n = 1
tools and the practice of change processes	[47]	n = 1
principles of high-quality project management	[120]	n = 1
principles of organizational and/or leadership development, and organizational strategic planning	[57]	n = 1
the principles of professional development	[118]	n = 1

**Facilitating EBP in general**	**Publication IDs**	**Number of publications**

ISPs have knowledge about…		
what characterizes (good) research	[56] [64] [101] [105] [106]	n = 5
the key principles of EBP	[94] [106] [107]	n = 3
knowledge translation	[48] [51] [98]	n = 3
general principles of high-quality practice	[50] [74] [121]	n = 3
how to source evidence	[101] [105] [106]	n = 3
how to conduct an evaluation	[57] [104]	n = 2
evidence synthesis	[56]	n = 1
how to select an RSI	[59]	n = 1
