# Supplementary material for: Knowledge and attitudes of Implementation Support Practitioners—Findings from a systematic integrative review
Source: PLoS One. 2022 May 11;17(5):e0267533. doi: 10.1371/journal.pone.0267533 (PMC9094539; doi:10.1371/journal.pone.0267533)
Supplement: S5 Appendix — The publication IDs indicate the corresponding articles in which the descriptors were identified. (PDF) [file pone.0267533.s005.pdf]

S5 Appendix: Overview of descriptors identified in the included studies categorized in the seven attitude themes (*professional, motivated / motivating / encouraging / empowering attitude, empathetic / respectful / sensitive attitude, collaborative / inclusive attitude, authentic attitude, creative / flexible / innovative / adaptive attitude, frank / direct / honest attitude*). The publication IDs indicate the corresponding articles in which the descriptors were identified.

**Attitudes**<br><br>*ISPs have a … attitude.*	**Publication references**	**Number of Publications**
**Professional**		**N = 28**
*Professional**	[82] [92] [100] [108]	n = 4
Responsive	[84] [85] [86] [91] [95] [99] [100] [108] [114] [118] [119] [123]	n = 12
Focused	[85] [89] [90] [100] [108] [113] [121] [122] [125]	n = 9
Credible	[91] [93] [99] [101] [106] [119] [121]	n = 7
Resilient	[84] [101] [106] [110] [113] [121]	n = 6
Perseverant	[101] [113] [121]	n = 3
Clear	[108] [115] [118]	n = 3
Research-minded	[98] [106]	n = 2
Persistent	[100] [110]	n = 2
Consistent	[103] [119]	n = 2
Proactive	[84]	n = 1
Autonomous	[84]	n = 1
Neutral	[84]	n = 1
Accurate	[103]	n = 1
Self-efficient	[111]	n = 1
Committed	[113]	n = 1
Patient	[92]	n = 1
Timely	[119]	n = 1
Discrete	[119]	n = 1

Reliable/dependable	[121]	n = 1
Intentional	[123]	n = 1
Accountable	[125]	n = 1
**Motivated / motivating / encouraging / empowering**		**N = 25**
*Motivated / motivating / encouraging / empowering**	[83] [85] [87] [89] [92] [94] [97] [99] [100] [101] [102] [103] [104] [106] [107] [108] [111] [113] [116] [118] [119] [120] [121] [122] [123]	n = 25
**Empathetic / respectful / sensitive**		**N = 20**
*Empathetic / respectful / sensitive**	[82] [85] [87] [90] [92] [95] [100] [101] [104] [106] [108] [110] [112] [118] [119] [120] [122] [123] [124] [125]	n = 20
**Collaborative / inclusive**		**N = 19**
*Collaborative / inclusive**	[85] [92] [94] [95] [96] [97] [98] [100] [101] [106] [108] [111] [112] [117] [118] [119] [123] [124] [125]	n = 19
**Authentic**		**N = 13**
*Authentic**	[81] [85] [95] [96] [98] [105] [108] [109] [113] [115] [119] [123]	n = 12
Self-aware	[121]	n = 1
**Creative / flexible / innovative / adaptive**		**N = 9**
*Creative / flexible / innovative / adaptive**	[86] [88] [98] [101] [106] [111] [115] [116] [117]	n = 9
**Frank / direct / honest**		**N = 5**
*Frank / honest / direct**	[81] [92] [113] [118] [122]	n = 5

* not further specified
