# Supplementary material for: Knowledge and attitudes of Implementation Support Practitioners—Findings from a systematic integrative review
Source: PLoS One. 2022 May 11;17(5):e0267533. doi: 10.1371/journal.pone.0267533 (PMC9094539; doi:10.1371/journal.pone.0267533)
Supplement: Attachment — Submitted filename: Response to Reviewers.docx [file pone.0267533.s006.docx]

**Rebuttal Letter – PONE-D-21-27502**

Manuscript: Knowledge and attitudes of Implementation Support Practitioners - Findings from a systematic integrative review

Dear Editor and Reviewers,

We appreciate the comments from the reviewers and the opportunity to submit a revised version. We incorporated the reviewers’ suggestions and believe that the comments highly contributed to the quality of this manuscript. Responses to your specific comments are detailed below.

**Reviewer #1:**

The authors have used a systematic integrative approach to describe the knowledge and attitude of Implementation Support Practitioners. The work builds on previous works by the authors. The introduction section is well done and clearly highlights the gap in consolidation in knowledge and attitudes aspects in Implementation area. The methods, results and discussion are well described.

The potential drawbacks of the study like possibility of omission of key studies, authors stating number of articles describing knowledge and attitude rather than number of ISPs have all been described very well in the study limitations area.

Authors have mentioned the seven attitude thematic area in the abstract, these are not numbered like it appears on page 23 of 55. I advise that these thematic areas are numbered in the abstract

Response: Thank you very much for this suggestion. We have included enumeration in the abstract and agree that this enhances readability.

**Reviewer #2:**

The authors describe the knowledge and attitudes of Implementation Support Practitioners (ISPs) through a systematic review. The content of the work is important and generally well presented but the methodology of the review is unclear.

Please find below my comments:

1. I find the conclusion vague and not really useful in answering the aim of the review which was to present the knowledge and attitudes of ISPs. Could the authors please revise the conclusion by possibly highlighting the key knowledge and attitude themes?

Response: Thank you for this comment. We added a few sentences to the conclusion to highlight the key knowledge and attitude themes identified in this review (p. 30, l. 655 – 661).

2. The methodology section could be improved as follows:
a. Please define what is meant by an integrative review in the methods section. I struggled to understand what is meant by an integrative until I read the limitations section. In addition please clarify why this is not a scoping review?
b. Please report the search section according to PRISMA for literature searches ;PRISMA-S(https://www.equator-network.org/reporting-guidelines/prisma-s/)
c. Sources searched for literature were broad including social media. Which social media platforms were searched?
d. Were there any limitations in the search with regard to publication date, language, type of publications?
e. Please state the inclusion and exclusion criteria for studies included in the review. What type of studies were eligible? Type of populations and geographical settings? Type of Intervention or concept? Type of Outcomes?
f. Knowing the type of eligible studies will help understand why only randomized trials were included for quality assessment.
g. How were the searches, data extraction and quality assessment done? Was an systematic review application such as covidence used to conduct the searches? How many reviewers did the study selection and data extraction? Was study selection done independently?
h. Was the data extraction form piloted and standardized? Which software or online platform was used to conduct data extraction?
i. It would be helpful to mention a priori in the methods section how the quality assessment was done. More details about the tool used for quality assessment, criteria for scoring?
j. Being a review, please also state in the analysis section that results were synthesized narratively/qualitatively. This will prevent further queries about quantitative results or analysis.

Response: Thank you for your comments regarding the methodology section. Please find below our remarks to the points you raised.

Re a. We included more details about the integrative review as a knowledge synthesis method (p. 8, l. 177-178). Our review provides more than an orientation and mapping of the role and attributes of ISPs in the field but rather integrates diverse data sources on ISP roles to develop a holistic understanding of the concept. That differentiates our systematic integrative review from a scoping review.

Re b. In addition to the general PRISMA checklist we have already used, we filled in the PRISMA-S checklist and attached it in the S2 Appendix.

Re c. We have included the social media channels that we have used for our literature search (p. 9, l. 203).

Re d. We have added a table on inclusion and exclusion criteria that includes the language and type of publication (p. 10-11). No limitations were defined for publication dates.

Re e. We have added a table on inclusion and exclusion criteria (p. 10-11).

Re f. We fully agree (also see e.).

Re g. We used the platform *Covidence* to screen the literature, this detail was added to the manuscript (p. 9, l. 187). All included articles were uploaded to *Dedoose* to code the data, as stated in the “Data analysis” subsection (p. 12, l. 222). The subsections “Literature search” and “Data evaluation” provide information on how the search and quality assessment (also see i.) were performed, respectively. We have included a paragraph on the constitution of the research team in the beginning of the methodology section (p. 9, l. 183-187).

Re h. The data extraction form was standardized but not systematically piloted. We have used the platform *Covidence* to screen the literature and *Dedoose* to code and analyse the data, as stated in the “Data analysis” subsection (p. 12, l. 222).

Re i. We provided more detailed information on the quality assessment and the framework used for this (Hodder et al., 2014) in the “Data evaluation” subsection (p. 11, l. 211-219).

Re j. We included this detail in the “Data analysis” section (p. 13, l. 243-244).

3. Results section
a. I dont find Table 1 helpful. A table of main study characteristics if applicable is better. Some information about the studies is more useful than listing the references only. Else that table can be moved to the appendices.
b. Figure 1 flow chart. Please specify in the first text box under search that these were electronic databases.
c. The results of the quality assessment are not clearly presented in the manuscript.

Response: Thank you for these helpful comments. We included an overview of the main study characteristics in S3 Appendix. This overview also indicates if a particular article reported on knowledge, or attitudes, or both, which was previously presented in Table 1. Table 1 was therefore removed from the manuscript. Figure 1 was amended as requested. We rephrased the paragraph on the quality assessment and highlighted the reference to S1 ERA, where the results of the quality assessment are presented.
