# Supplementary material for: Knowledge and attitudes of Implementation Support Practitioners—Findings from a systematic integrative review
Source: PLoS One. 2022 May 11;17(5):e0267533. doi: 10.1371/journal.pone.0267533 (PMC9094539; doi:10.1371/journal.pone.0267533)
Supplement: Attachment — Submitted filename: Response to Reviewers.docx [file pone.0267533.s007.docx]

**Rebuttal Letter – PONE-D-21-27502**

Manuscript: Knowledge and attitudes of Implementation Support Practitioners - Findings from a systematic integrative review

Dear Eleanor Ochodo,

We appreciate your comments and the opportunity to submit a revised version. Responses to your specific comments are detailed below.

**Additional Editor Comments:**

Thank you for satisfactorily addressing most of the previous reviewer comments. Please address the following minor comments:

1. In page 9, the authors state that " The researchers double-screened the titles, abstracts, and full-texts independently....." . However, this statement is only about study selection. Please also specifically state that data extraction/evaluation were done independently. The Prisma checklist in the appendix states that how risk of bias assessment (data evaluation in your case) was done is detailed in page 9. But that is not so.

Response: Thank you for this comment. To clarify this section, we have moved the information on the screening procedure (“The researchers double-screened the titles, abstracts, and full-texts independently using the platform *Covidence*. Conflicts were solved by a third member of the team.”, p. 9, l. 189) to the section ‘Data Evaluation’, p. 11, l. 216/217. We added information on the data extraction, as requested, to this paragraph:

“Data was extracted from the included studies by using a standardized data extraction form consisting of 19 items (i.e., study design and aim, method, geography, sector, setting, sampling strategy, sample size, clinical intervention, ISP information, outputs, and outcomes). Data from each included study was extracted by one research team member. The quality of the data extraction was assured by the lead author.” (p. 11, l. 217 - 222)

The PRISMA Checklist (appendix 2) hast been updated accordingly.

2. Also please add in the Abstract methods section that quality assessment of included studies was done and with which tool.

Response: Information regarding the quality assessment has been added as requested to the abstract (p. 2, l. 39 – 42):

“Article screening was performed independently by two researchers, and data from included studies were extracted by a member of the research team and quality-assured by the lead researcher. The quality of included RCTs was assessed based on a framework by Hodder and colleagues.”
